# Lymphocyte Exhaustion in AML Patients and Impacts of HMA/Venetoclax or Intensive Chemotherapy on Their Biology

**DOI:** 10.3390/cancers14143352

**Published:** 2022-07-10

**Authors:** Dmitry Zhigarev, Asya Varshavsky, Alexander W. MacFarlane, Prathiba Jayaguru, Laura Barreyro, Marina Khoreva, Essel Dulaimi, Reza Nejati, Christina Drenberg, Kerry S. Campbell

**Affiliations:** 1 Blood Cell Development and Function Program, Institute for Cancer Research, Fox Chase Cancer Center, Philadelphia, PA 19111, USA; dmz4001@med.cornell.edu (D.Z.); alexander.macfarlane@fccc.edu (A.W.M.IV); 2 Department of Immunology, Pirogov Russian National Research Medical University, Moscow 117997, Russia; mv@rsmu.ru; 3 Department of Bone Marrow Transplant and Cellular Therapies, Fox Chase Cancer Center, Philadelphia, PA 19111, USA; asyanina.varshavskyyanovsky@fccc.edu; 4 Oncology Translational Research, Janssen R&D, Spring House, PA 19477, USA; pjayagur@its.jnj.com (P.J.); lbarreyr@its.jnj.com (L.B.); cguttke@its.jnj.com (C.D.); 5 Department of Pathology, Fox Chase Cancer Center, Philadelphia, PA 19111, USA; esse.al-saleem@towerhealth.org (E.D.); reza.nejati@fccc.edu (R.N.)

**Keywords:** AML, immunopharmacology, venetoclax, hypomethylating agents, decitabine, 5-azacytidine, intensive chemotherapy, T cell exhaustion, immune checkpoint receptors, adaptive NK cells

## Abstract

**Simple Summary:**

Patients with acute myeloid leukemia (AML) are routinely treated with either intensive chemotherapy or DNA hypomethylating agents (HMA) in combination with the Bcl-2 inhibitor, venetoclax. While both treatment regimens are highly cytotoxic to the aggressive AML tumor cells, they are also toxic to immune cells. Therefore, we sought to establish the detrimental impacts of these therapies on lymphocytes and their recovery over time in AML patients. Even prior to treatment initiation, the patients were found to have exhausted lymphocytes in peripheral blood, and additional signs of exhaustion were noted after treatment with HMA/venetoclax. In fact, the lymphocytes were still suppressed for two to three months after the initiation of induction therapy. Furthermore, T cells in a subset of patients subsequently found to be resistant to venetoclax therapy exhibited a higher expression of perforin and CD39 and more pronounced IFN-γ production.

**Abstract:**

Acute myeloid leukemia (AML) is an aggressive malignancy that requires rapid treatment with chemotherapies to reduce tumor burden. However, these chemotherapies can compromise lymphocyte function, thereby hindering normal anti-tumor immune responses and likely limiting the efficacy of subsequent immunotherapy. To better understand these negative impacts, we assessed the immunological effects of standard-of-care AML therapies on lymphocyte phenotype and function over time. When compared to healthy donors, untreated AML patients showed evidence of lymphocyte activation and exhaustion and had more prevalent CD57^+^NKG2C^+^ adaptive NK cells, which was independent of human cytomegalovirus (HCMV) status. HMA/venetoclax treatment resulted in a greater fraction of T cells with effector memory phenotype, inhibited IFN-γ secretion by CD8^+^ T cells, upregulated perforin expression in NK cells, downregulated PD-1 and 2B4 expression on CD4^+^ T cells, and stimulated Treg proliferation and CTLA-4 expression. Additionally, we showed increased expression of perforin and CD39 and enhanced IFN-γ production by T cells from pre-treatment blood samples of venetoclax-resistant AML patients. Our results provide insight into the lymphocyte status in previously untreated AML patients and the effects of standard-of-care treatments on their biology and functions. We also found novel pre-treatment characteristics of T cells that could potentially predict venetoclax resistance.

## 1. Introduction

Acute myeloid leukemia (AML) is a deadly malignancy of myeloid cell progenitors with a high relapse rate [1]. In the US, the median age at diagnosis is 68 years, with 10–15% 5-year survival for patients >60 years and 30–35% for younger patients [2]. About 80% of younger patients can achieve complete response (CR) with timely treatment [3], but most will eventually relapse without allogeneic hematopoietic stem cell transplantation (allo-HSCT) [3,4].

Eligible AML patients for intensive induction chemotherapy receive the “7+3” treatment regimen [2], consisting of seven days continuous infusion of cytarabine, along with anthracycline during the first three days. This treatment scheme has some variations, notably Vyxeos (CPX-351), which consists of liposome-embedded cytarabine and daunorubicin and has improved overall survival (OS) in older patients with secondary AML [5,6]. However, these treatments are still highly toxic, causing significant hematopoietic cell depletion.

The hypomethylating agents (HMAs), 5-azacytidine and decitabine, were FDA-approved for treating myeloid malignancies. Their mechanism of action remains unclear but related to the inhibition of DNA methyltransferases, thereby hypomethylating DNA and inducing expression of silenced gene loci, including tumor suppressor genes [7]. OS for older AML patients undergoing HMA treatment is improved compared to intensive chemotherapy (IC) [8,9]. Thus, HMAs are more commonly used to treat elderly or debilitated patients who cannot tolerate conventional IC [10]. Nonetheless, the high relapse rates after “7+3” or HMA treatments demonstrate an urgent need for better therapeutic strategies.

Several novel targeted agents were recently approved to treat AML, such as the B cell lymphoma 2 (Bcl-2) inhibitor, venetoclax, which is commonly administered in combination with HMAs due to higher efficacy compared to HMAs alone [11,12].

Immunotherapy is another option to consider for treating AML, but immune status is already compromised in untreated patients [13,14,15], and chemotherapies can further negatively affect the activity, survival, and phenotypic features of immune cells [16,17,18]. Here, we assessed the phenotype and function of lymphocytes from peripheral blood of AML patients before and at various times after treatment with either the combination of HMA and venetoclax or IC. The data shed light on the baseline features of lymphocytes in untreated AML patients and the impacts of standard treatments on these features. We observed lymphocyte exhaustion and a high incidence of adaptive NK cells in untreated AML patients, significant long-term depletion and changes in receptor expression in response to both treatments, and novel characteristics of T cells in patients with venetoclax resistance.

## 2. Materials and Methods

### 2.1. Donor Characteristics

We studied 32 AML patients prior to initial therapy or without prior anticancer therapy for at least 2 years (details in Supplementary Table S1). In total, 17 patients were treated with IC, mainly “7+3” or modifications, and 15 were treated with the combination of HMA and venetoclax. Patient ages varied from 21–82 years (mean = 60.4 ± 14.5), with the average age of the HMA/venetoclax-treated group at 67.4 ± 12.5 years and chemotherapy-treated at 54.2 ± 13.4 years (Supplementary Figure S1A), consistent with the use of HMAs in older patients. Healthy donors (HDs; *n* = 11) with a similar age to the experimental group (mean = 63.5 ± 7.1 years) were recruited. The study was conducted according to the ethical standards of the Helsinki Declaration and approved by our institutional review board (#17-8010). Written consent was obtained prior to obtaining blood samples, and samples were de-identified before release to the laboratory.

### 2.2. Patient Therapies and Blood Sampling

Peripheral blood was drawn into Vacutainer EDTA tubes (BD—Becton Dickinson, Franklin Lakes, NJ, USA). The first sample was drawn before therapy and designated “before treatment”. This baseline sample was also compared with blood from HDs. Four patients were withdrawn after the first blood sample due to critical health conditions. Therapeutic interventions began immediately after the first sample. Patients treated with IC received an initial course of seven days cytarabine (100–200 mg/m^2^) and three days of idarubicin (12 mg/m^2^). Vyxeos was given on days 1, 3, and 5 (daunorubicin 44 mg/m^2^, cytarabine 100 mg/m^2^). HMA/venetoclax patients were initially treated with five days decitabine (20 mg/m^2^) or five to seven days 5-azacytidine (75 mg/m^2^). A second blood sample (“early recovery”) was obtained at the time of adequate lymphocyte recovery for analysis (Supplementary Figure S1B). A previous report established median lymphocyte recovery for IC at 18 days after the end of therapy (range = 12–29 days) [19], which is consistent with the second blood sample in our IC-treated patients at a median of 20 days after the end of induction therapy (range = 10–29 days; note that days after the first sample are plotted on Supplementary Figure S1B). In the HMA/venetoclax-treated group, the second blood sampling was drawn a median of 15.5 days after the end of the initial HMA cycle (range = 10–24 days). A third sample (“before 2nd treatment”) was drawn before the start of the second therapy cycle or at a comparable time if patients did not receive a second therapy (Supplementary Figure S1B). For patients treated with IC, the third sample was drawn a median of 43 days after the first sample (range = 31–97 days); for HMA-treated patients, the median was 35 days (range = 33–46 days). The last blood sample (“after 2nd treatment”) was obtained during long-term recovery after the second therapy, often just before allo-HSCT (Supplementary Figure S1B). For the IC-treated group, this final sample was drawn at a median of 69 days (range = 42–106 days) after the first sample, and the median timepoint for HMA/venetoclax-treated patients was 60 days (range = 46–71 days).

### 2.3. Peripheral Blood Mononuclear Cells (PBMCs) Isolation and Cell Culture

PBMCs from AML patients and HDs were isolated using Lymphoprep (StemCell Technologies, Vancouver, BC, Canada) density gradient centrifugation. The PBMC interface was resuspended in Ca^2+^/Mg^2+^-free Dulbecco’s phosphate-buffered saline, washed, and adjusted to 10 × 10^6^ cells/mL in RPMI-1640 medium without Phenol Red. The EBV-transformed 721.221 target B cell line was cultured in RPMI-1640 medium with 15% heat-inactivated fetal bovine serum (FBS), 2 mM L-glutamine, 10,000 U/mL streptomycin/penicillin, and 10 mM HEPES in 7% CO_2_ at 37 °C. The 7% CO_2_ level is standardly used in our laboratory to provide better culture conditions for NK cells.

### 2.4. Flow Cytometry

Our panel included antibodies to more than 40 surface and intracellular markers, as shown in Supplementary Table S2. PBMCs (1 × 10^6^ cells/100 μL) were incubated with antibodies for 20 min on ice and washed twice with washing buffer (Hanks’s Balanced Salt Solution Ca^2+^/Mg^2+^ free (Fox Chase Cancer Center Cell Culture Facility, Philadelphia, PA, USA), 1% Heat-Inactivated FBS, 0.9% NaN_3_), with propidium iodide (100 ng/mL) in the last wash to detect dead cells. For intracellular protein staining, Ghost Red 710 Viability Dye (TONBO Biosciences, San Diego, CA, USA) was added prior to permeabilization. PBMCs (1 × 10^6^ cells/100 μL) were then incubated with fixation/permeabilization buffer (Invitrogen/ThermoFisher, Pittsburgh, PA, USA, #00-5523-00) for 25 min on ice. Then, antibodies to intracellular biomarkers (perforin, FoxP3, Ki67, CTLA-4, and BLIMP-1) were added for 30 min on ice, and cells were rinsed twice with the kit wash buffer. Analysis was performed on a 4-laser BD ARIA II flow cytometer using a configuration with 14 fluorescence channels, as previously described [20]. The cytometer was CLIA- and CAP-certified, and calibration of the cytometer was performed daily using BD CS&T Research Beads in the same manner throughout the study. Compensation and PMT voltage settings were consistent for acquisition of all samples, as optimized at the beginning of the project based upon analysis of unstained, single-stained, and multi-stained PBMC samples. BD FACSDiva software v.8.0.1 was used to record data, and .fcs files were processed using FlowJo v10 Software (BD). Gating strategies and representative staining profiles of major biomarkers are shown in Supplementary Figures S2–S4. Distinct immune parameters were quantified from each blood sample as the percent of a cell subset expressing a marker or the geometric mean fluorescence intensity (GMFI) of expression. Data were represented as a percentage of marker-positive cells when the composition of a given subpopulation of cells expressing a marker(s) was studied (e.g., NKG2C^+^CD57^+^ adaptive NK cells, KIR^+^ mature NK cells, or CD39^+^ exhausted cells) and as GMFI when the focus of an experiment was to investigate the expression level of a marker. Exceptions to this rule are 2B4, presented as a percentage on CD4^+^ T cells due to the high statistical significance in the change of the percentage of expressing cells, and NKp44, presented as a proportion of positive NK cells due to a relatively low signal that limits detection of change using the GMFI parameter.

### 2.5. IFN-γ Secretion Assay

An IFN-γ secretion assay (Miltenyi Biotec, Bergisch Gladbach, Germany) was used to assess T and NK cell function. Aliquots of PBMCs (1 × 10^6^ in 100 µL) were treated with: (1) CytoStim (Miltenyi; 2 µL/tube) + IL-2 (50 U/mL) to stimulate T cells; (2) 721.221 cells (1 × 10^6^) + rituximab (1 µg/mL) to simulate ADCC conditions for NK cell stimulation; or (3) IL-2 (50 U/mL); and (4) PBMCs alone as controls. Aliquots were incubated for 2.5 h in 7% CO2 at 37 °C. Cells were washed with washing buffer and cultured 10 min on ice with 10 μL of bivalent anti-CD45/anti-IFN-γ antibody (IFN-γ Catch Reagent, Miltenyi) to capture locally secreted IFN-γ on leukocyte surfaces. RPMI-1640 medium (7 mL; 37 °C) was added and shaken 45 min in 7% CO_2_ at 37 °C. Then, cells were stained with fluorophore-conjugated antibodies toward IFN-γ, CD3, CD4, CD8, CD56, and NKp80, washed twice, and stained with propidium iodide (100 ng/mL). Cytometric analysis and data processing were performed as above. The results were presented as the percent of IFN-γ-producing cells, as suggested by the manufacturer.

### 2.6. Cytokine Multiplex Array

Plasma samples were collected and stored at −80 °C within 2 h after collection. Proinflammatory cytokine (IL-2, IL-4, IL-10, IL-8, IL-6, IL-12p70, TNF-α, IL-13, IFN-γ, IL-1β) levels in plasma were measured using Meso Scale Discovery (MSD, Mesoscale Diagnostic, Rockville, MD, USA) following the manufacturer’s protocol. Briefly, plasma samples and calibrators were added to an MSD 96-well plate coated with the corresponding capture antibodies in technical duplicates. Plates were incubated for 2 h, and unbound analyte was removed using an automated plate washer (Biotek ELx405 with a Biostack 3 plate stacker, Agilent, Santa Clara, CA, USA). SULFO-TAG–conjugated detection antibodies were added to the plates, and plates were incubated for 2 h and then washed to remove unbound detection antibody. Read buffer (manufacturer provided) was added, and plates were analyzed with an MSD instrument (MesoTM Sector S 600). Cytokine levels were determined using Mesoscale Discovery Workbench 4.0.

### 2.7. Human Cytomegalovirus (HCMV) Testing

Anti-HCMV IgG ELISA Kit (Abcam, Cambridge, UK) was used to determine HCMV serological status of patients. Frozen plasma samples were thawed at RT and diluted 1:100 with a diluent reagent. All samples and controls were assayed in duplicate in accordance with the manufacturer’s protocol. Samples were considered HCMV-seropositive if the absorbance value was greater than 10% over the cut-off value.

### 2.8. Statistical Analysis

Calculations, statistics, and plotting were performed using Excel (Microsoft 365, v. 2205, Redmond, WA, USA), GraphPad Prism 9 (GraphPad Software, San Diego, CA, USA), MatLab v9.6 (MathWorks Inc., Natick, MA, USA), and R Studio v.1.3.1093-1—2021.09.0 (RStudio, PBC, Boston, MA, USA) software. The Wilcoxon signed-rank test was used to establish statistical significance between parameters at different timepoints. The nonparametric Mann–Whitney U test was used to compare sets of AML patients and HDs. For correlation analysis, the Spearman correlation criterion was used. For all tests, *p*-values < 0.05 were considered statistically significant.

## 3. Results

### 3.1. Lymphocytes from Untreated AML Patients Show an Exhausted Phenotype Compared to Healthy Donors

**Figure 1:**
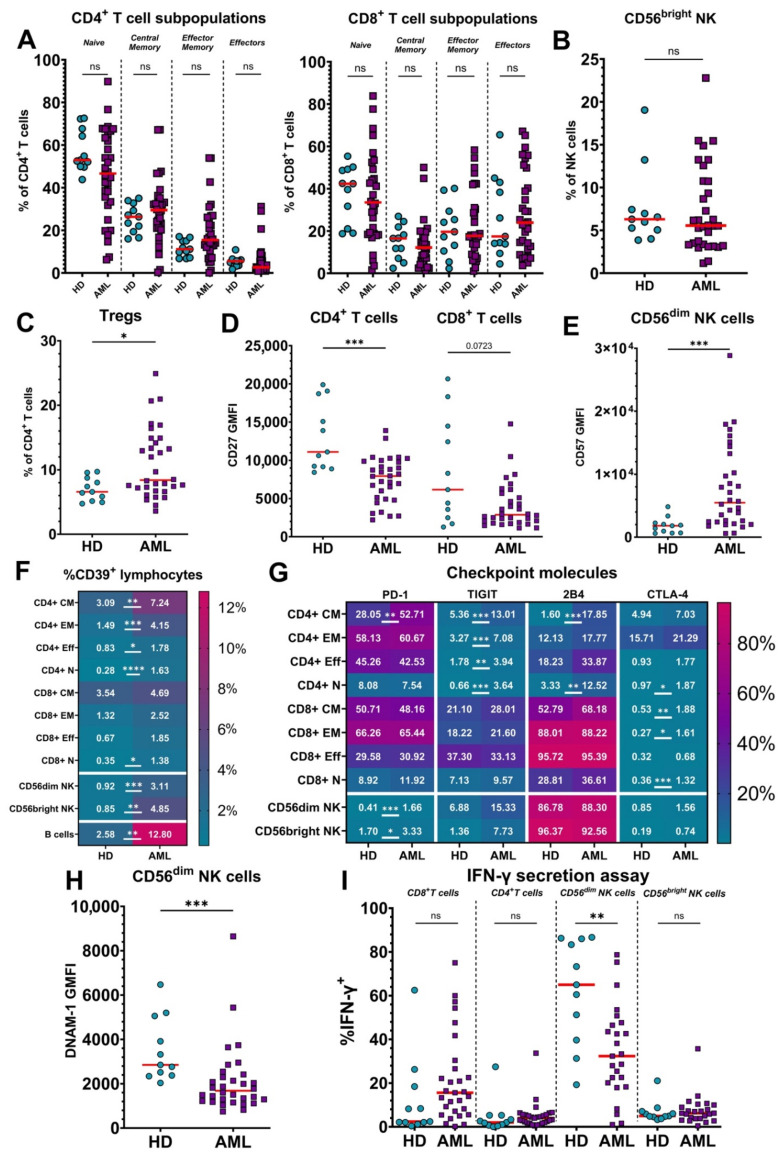
Comparison of the characteristics of lymphocytes from untreated acute myeloid leukemia (AML) patients and healthy donors. Lymphocytes of 32 AML patients and 11 healthy donors (HDs) were examined to identify differences in their phenotype and function. (**A**) Percentages of subsets of CD4^+^ (left) and CD8^+^ (right) T cells were measured by flow cytometry by staining CD45RA and CD62L; medians are marked by red bars. HDs are represented by blue circles, AML patients by purple squares. (**B**) Percentage of immature CD56^bright^ NK cells among total NK cells from HDs and untreated AML patients. (**C**) Percentage of CD25^high^CD127^low^ Tregs among CD4^+^ T cells from HDs and untreated AML patients. (**D**) Geometric Mean Fluorescence Intensity (GMFI) of CD27 expression on CD4^+^ and CD8^+^ T cells from HDs and untreated AML patients. (**E**) GMFI of CD57 expression on CD56^dim^ NK cells from HDs and untreated AML patients. (**F**) Proportion of CD39 expressing lymphocytes from HDs and untreated AML patients. The data are shown as a heatmap of median percentage expression of CD39 with scale at the right. Rows represent different lymphocytic populations: CM—central memory, EM—effector memory, Eff—effector, N—naïve. (**G**) Median percentages of HDs and untreated AML patient lymphocyte subsets expressing immune checkpoint molecules PD-1, TIGIT, 2B4, and CTLA-4, as described for panel F. (**H**) GMFI of DNAM-1 expression on mature CD56^dim^ NK cells from untreated AML patients. (**I**) Comparison of the percentages of major T and NK cell subsets secreting IFN-γ from HDs and AML patients measured after 2.5 h of in vitro stimulation. The reduced number of AML patients in both assays is due to inadequate numbers of lymphocytes in some untreated blood samples. Mann–Whitney nonparametric U test was used for statistical analysis. ns—*p* > 0.05; *—*p* < 0.05; **—*p* < 0.01; ***—*p* < 0.001; ****—*p* < 0.0001. Actual *p*-value is specified in cases when it is close to 0.05.

First, we compared baseline flow cytometry data from untreated patients (*n* = 32) with that of healthy donors (HDs; *n* = 11) using the antibody panel and IFN-γ secretion assay described in Materials and Methods. While the distribution of percentages of distinct subpopulations (naïve, central memory, effector memory, and effectors) within the CD4^+^ and CD8^+^ T cell compartments were tighter for HDs, we found no significant differences between AML patients and HDs, although some individual patients exhibited substantial shifts in ratios of T cell subsets (Figure 1A). The percentage of immature CD56^bright^ NK cells among total NK cells was also similar in the AML patients compared to HDs (Figure 1B). In contrast, we observed an increase in the proportion of CD4^+^CD25^high^CD127^low^ regulatory T cells (Tregs) among total CD4^+^ T cells in the peripheral blood of AML patients (Figure 1C).

Evidence for potentiated T and NK cell maturation was observed in the AML patients, where the surface expression of CD27 was decreased on both CD4^+^ (significant) and CD8^+^ (trending) T cells compared to HDs (Figure 1D). Additionally, CD56^dim^ NK cells in patients showed greater expression of CD57, which marks terminal NK cell maturation (Figure 1E).

AML patients also showed upregulated expression of CD39 (Figure 1F) on B, NK, and CD4^+^ T cells, which indicates their exhaustion or immunosuppressive function [21,22,23,24]. Furthermore, various subsets of T and NK cells from AML patients expressed higher levels of the inhibitory checkpoint markers, PD-1, TIGIT, 2B4, and CTLA-4 (Figure 1G). Notably, TIGIT was increased in all CD4^+^ T cell subsets, as was CD39, whereas PD-1 and 2B4 were more selectively upregulated in central memory CD4^+^ T cells. Although the difference of TIGIT expression on NK cells was not statistically significant, the expression of the activating receptor DNAM-1, which shares the same ligands as inhibitory TIGIT, was downregulated on CD56^dim^ NK cells in the AML patients (Figure 1H). To functionally test the activity of T and NK cells, we performed an IFN-γ secretion assay. The secretion of IFN-γ by CD56^dim^ NK cells under ADCC-inducing conditions was significantly suppressed in AML patients compared to HDs, again consistent with the exhausted phenotype (Figure 1I). Paradoxically, when stimulated with CytoStim, CD8^+^ T cells from many AML patients showed a trend toward more IFN-γ secretion compared to healthy donors, although this was not statistically significant.

### 3.2. Adaptive NK Cells Are Significantly Increased in Blood of AML Patients, Independent of HCMV Serotype

**Figure 2:**
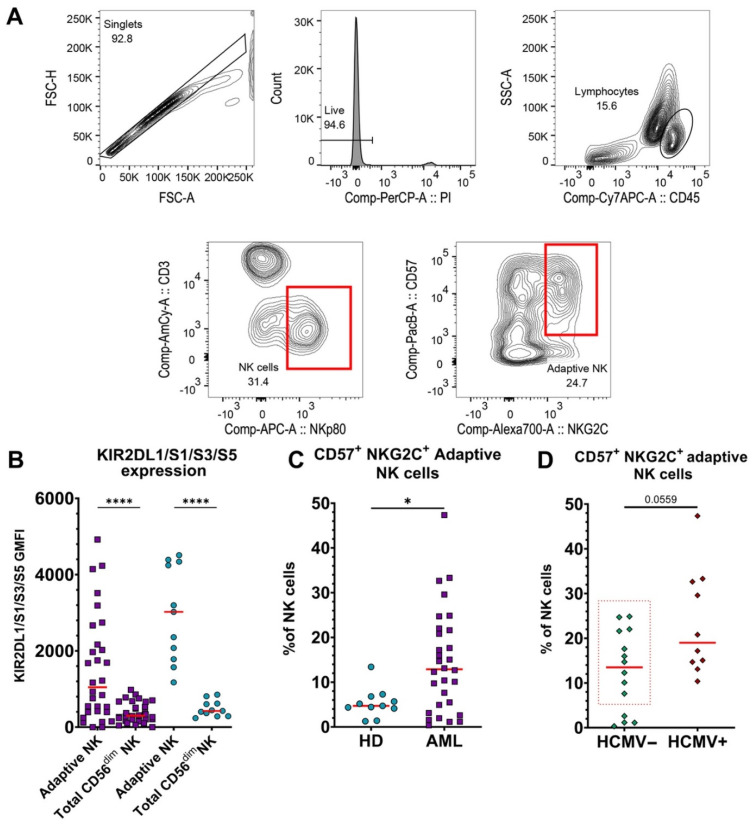
CD57^+^NKG2C^+^ adaptive NK cells are prevalent in the peripheral blood of AML patients. PBMCs from 30 untreated AML patients and HDs were assessed for adaptive NK cells. A reduced number of AML patients is due to low numbers of NK cells in two untreated blood samples. (**A**) Gating strategy for adaptive NK cells. First, single cells were selected in a forward scatter area (FSC-A) vs. forward scatter height (FSC-H) plot. Then, living cells were selected as propidium iodide (PI)-negative. Lymphocytes were identified as CD45^+^SSC^low^ cells in a CD45 vs. side scatter area (SSC-A) dot plot. NK cells were defined as CD3^-^NKp80^+^ lymphocytes. Finally, CD57^+^NKG2C^+^ NK cells were identified as adaptive NK cells. (**B**) GMFI of KIR2DL1/S1/S3/S5 expression on adaptive NK cells vs. total mature CD56^dim^ NK cells from AML patients (purple squares) and HDs (blue circles). (**C**) Comparison of % CD57^+^NKG2A^+^ adaptive NK cells from peripheral blood of HDs and AML patients. (**D**) Percentage of CD57^+^NKG2C^+^ adaptive NK cells in blood of untreated AML patients (*n* = 24) that tested negative for HCMV (HCMV^−^) vs. positive (HCMV^+^). The red dotted rectangle emphasizes HCMV^–^ patients with significant percentages of adaptive NK cells. For panels (**B**–**D**), red lines represent medians. Mann–Whitney nonparametric U test was used for statistical analysis. ns—*p* > 0.05; *—*p* < 0.05; ****—*p* < 0.0001. A *p*-value is specified in cases when it is close to 0.05.

To assess adaptive NK cells, we gated CD3^-^NKp80^+^NKG2C^+^CD57^+^ lymphocytes [25] using the gating strategy shown in Figure 2A and representative examples from different donors are shown in Supplementary Figure S5. These cells also expressed higher levels of killer cell Ig-like receptors (KIRs) (Figure 2B and Supplementary Figure S1C), and the percentage of CD56^dim^ NK cells expressing KIRs negatively correlated with NKG2A expression (Supplementary Figure S1D), consistent with previous literature on the phenotype of adaptive NK cells [26]. The percentage of adaptive NK cells was greater in the blood of AML patients compared to HDs (Figure 2C), contributing to the increased CD57 expression (Figure 1E). Since the presence of adaptive NK cells was previously shown to correlate with human cytomegalovirus (HCMV) status [25], we assessed the serological status of the patients. In contrast to previous reports with non-cancer patients, ten out of fourteen HCMV-seronegative AML patients had significant proportions of adaptive NK cells in their blood (Figure 2D). Adaptive cells from AML patients also exhibited higher expression levels of CD56 and CD57, as compared to HDs, while levels of KIRs and DNAM-1 were significantly lower (Supplementary Figure S1E).

### 3.3. Both IC and HMA/Venetoclax Aggressively Deplete Lymphocytes, and HMA/Venetoclax Therapy Increases the Frequencies of T cells with an Effector Memory Phenotype

We next followed the AML patients through the course of two distinct treatment regimens: (1) 5-azacytidine or decitabine + venetoclax (“HMA/venetoclax” on figures) and (2) IC (“chemotherapy” on figures). The timing of the samples is detailed in Materials and Methods and shown in Supplementary Figure S1B.

**Figure 3:**
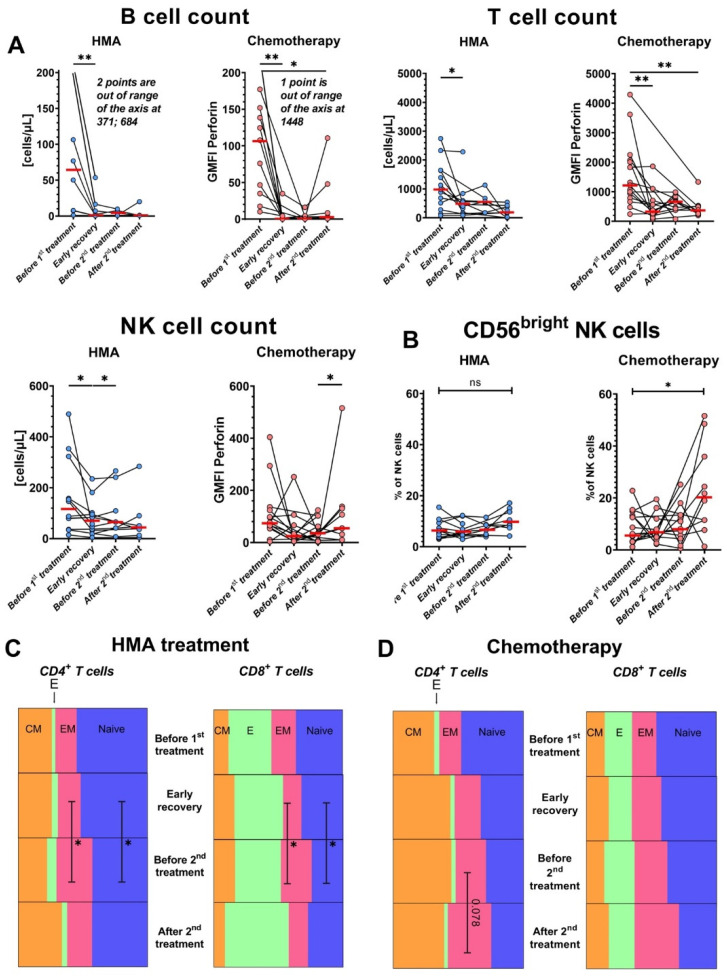
Both treatments deplete lymphocytes, and HMA/venetoclax therapy shifts T cells toward the effector memory phenotype. AML patients with >1 sample collected were divided into two groups based on the treatment scheme: (1) combination of HMA/venetoclax (“HMA”, blue circles) and (2) IC (“Chemotherapy”, pink circles). Red bars represent medians. (**A**) Changes in absolute numbers of lymphocytic populations over the course of treatments. (**B**) Percentage of immature CD56^bright^ NK cells among total NK cells during the courses of treatment with HMA (left) or IC (right). (**C**) Parts-of-whole diagrams showing changes in the median proportions of CD4^+^ (left) and CD8^+^ (right) T cells during the HMA treatment course. T cell subsets are: CM—central memory (orange), E—effector (green), EM—effector memory (pink), naïve (blue). Rows show timepoints from the top (“before treatment”) to the bottom (“after treatment”). (**D**) As in panel C, part-of-whole diagram showing changes in the proportion of T cell subsets during the course of intensive chemotherapy. Wilcoxon signed-rank test was used for statistical analysis. ns—*p* > 0.05; *—*p* < 0.05; **—*p* < 0.01.

To determine how these treatments affect the circulating lymphocytes of patients, we first measured absolute numbers of major lymphocyte subtypes along the course of therapy (Figure 3A). T, NK, and B cells were depleted in blood after initial therapy, especially B cells (median from 76.9 cells/µL to 1 cell/µL). NK cells were somewhat less sensitive to the therapies and showed some partial recovery by the final timepoint after chemotherapy, consistent with published data on their early recovery after patients receive HSCT [27]. The IC-treated patients selectively showed a significant increase in CD56^bright^ NK cells by the final “after treatment” timepoint (Figure 3B), but these samples were acquired at later timepoints than the final HMA/venetoclax samples (Supplementary Figure S1B). HMA/venetoclax increased the proportion of both CD4^+^ and CD8^+^ effector memory T cells at the expense of naïve T cells (Figure 3C). IC did not significantly impact percentages of T cell subpopulations, although CD4^+^ effector memory T cells showed a trending increase after the second treatment (Figure 3D).

### 3.4. HMA/Venetoclax Therapy Significantly Inhibits IFN-γ Production by CD8^+^ T Cells

**Figure 4:**
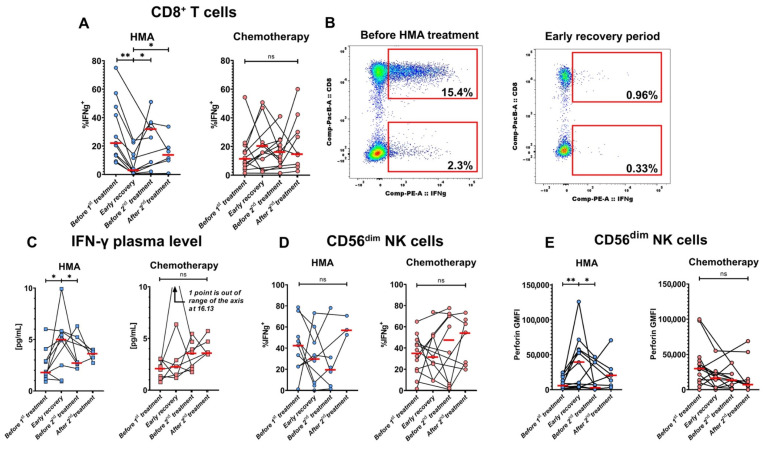
HMA/venetoclax treatment inhibits IFN-γ production by T cells and enhances perforin accumulation in cytotoxic lymphocytes. AML patients were divided into treatment groups: (1) HMA/venetoclax (“HMA”, blue circles, *n* = 12 for T cell assay, *n* = 10 for NK cell assay) and (2) IC (“Chemotherapy”, pink circles, *n* = 14 for T cell assay, *n* = 13 for NK cell assay). All patients with >1 sample collected were included in the plots. (**A**) Dynamics of % IFN-γ-secreting CD8^+^ T cells during HMA/venetoclax (left) and IC (right) treatments. (**B**) Representative flow cytometry dot plots of changes in frequencies of IFN-γ-secreting CD8^+^ T cells before treatment (left) and after the induction of HMA/venetoclax therapy (right) from patient #28 (see Supplementary Table S1). The clusters of cells displayed at the bottom of each panel are CD4^+^, and top clusters are CD8^+^. Numbers are percentages of total CD4^+^ and CD8^+^ T cells. (**C**) Dynamics of IFN-γ level in plasma from AML patients treated with HMA/venetoclax (left) and IC (right). (**D**) % IFN-γ-secreting CD56^dim^ NK cells during the courses of HMA/venetoclax (left) and IC (right). (**E**) Change in CD56^dim^ NK cell perforin GMFI under HMA/venetoclax treatment (left) and IC (right). Red bars represent medians. Wilcoxon signed-rank test was used for statistical analysis. ns—*p* > 0.05; *—*p* < 0.05; **—*p* < 0.01.

To understand therapy impacts on T and NK cell function, we performed an IFN-γ secretion assay after stimulation with CytoStim or ADCC-inducing conditions, respectively. We observed a strong inhibition of IFN-γ production by CD8^+^ T cells from patients after initial HMA/venetoclax therapy, but not IC (Figure 4A). The percentage of IFN-γ-producing T cells consistently decreased in all patients after initial HMA/venetoclax treatment and did not fully recover even by the last sample. A representative example of the potent suppression is shown in Figure 4B. In stark contrast, the concentration of IFN-γ in the plasma of the HMA/venetoclax-treated patients increased after the initial HMA/venetoclax treatment (Figure 4C). On the other hand, changes in production of IFN-γ by NK cells were highly variable between patients and not significant overall (Figure 4D).

### 3.5. Levels of Perforin Increase in NK Cells after HMA/Venetoclax Treatment

The intracellular level of perforin increased in cytolytic CD56^dim^ NK cells after initial treatment with HMA/venetoclax, but not IC (Figure 4E). The levels of perforin mostly returned to the baseline in subsequent timepoints. In contrast, levels of IFN-γ were not significantly altered in CD8^+^ T cells by either treatment.

### 3.6. PD-1 and 2B4 Surface Exposure Is Lowered on CD4^+^ T Cells from Patients Treated with HMA/Venetoclax

**Figure 5:**
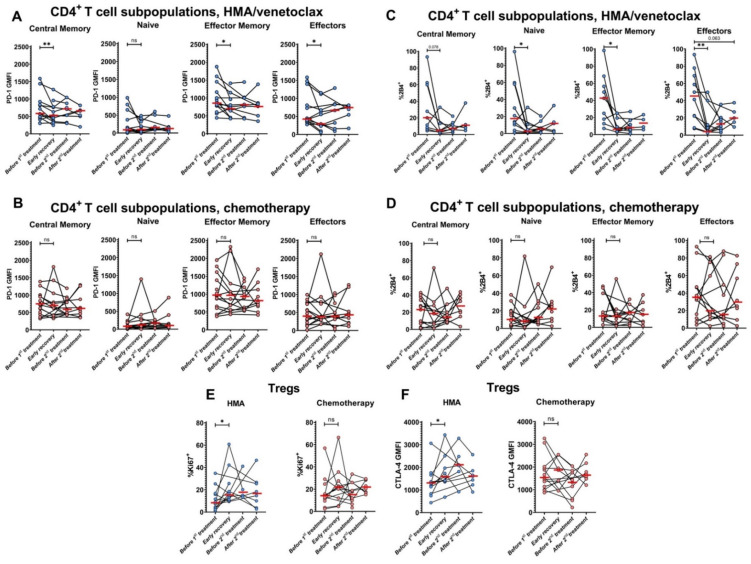
Initial course of HMA/venetoclax treatment reduces expression of checkpoint molecules PD-1 and 2B4 and enhances Treg proliferation and CTLA-4 expression. GMFI of PD-1 expression on CD4^+^ T cell subpopulations during treatment with HMA/venetoclax (**A**) or IC (**B**). (**C**,**D**) The changes in % CD4^+^ T cell subpopulations expressing 2B4 when treated with HMA/venetoclax (**C**) or IC (**D**). (**E**) Dynamics of the proportions of Ki67^+^ Tregs during the treatments. (**F**) GMFI of CTLA-4 expression on Tregs during the treatments. Red bars represent medians. Data are from patients with >1 sample collected. Blue circles are HMA/venetoclax, and pink circles are chemotherapy. Wilcoxon signed-rank test was used for statistical analysis. ns—*p* > 0.05; *—*p* < 0.05; **—*p* < 0.01. Actual p-value is specified in cases when it is close to 0.05.

We also examined the expression of the inhibitory checkpoint receptors in response to the therapies. PD-1 expression was decreased after the initial HMA/venetoclax treatment on all CD4^+^ T cell subpopulations except naïve (Figure 5A). In contrast, expression of PD-1 was not changed on any CD4^+^ T cell subset in IC-treated patients (Figure 5B). Similarly, patients treated with HMA/venetoclax showed decreased expression of 2B4 checkpoint receptor on all CD4^+^ T cell subpopulations, especially on CD4+ effector T cells (Figure 5C). Levels of 2B4 expression were also unchanged in the IC-treated group (Figure 5D). Interestingly, neither treatment impacted expression of either protein on CD8^+^ T cells.

### 3.7. Treg Proliferation and CTLA-4 Expression Are Enhanced after HMA/Venetoclax Therapy

To assess the status of Tregs, we measured their expression of Ki67 and CTLA-4 at different stages of treatment. We found that HMA/venetoclax enhanced the proportion of Tregs expressing Ki67, indicating that more of these immunosuppressive cells are proliferating (Figure 5E), although the percentage and absolute counts of Tregs were unchanged in the blood of the same cohort. The expression of CTLA-4 on Tregs was also increased after initial treatment with HMA/venetoclax (Figure 5F).

### 3.8. NK Cells Become Activated and Alter Receptor Expression after Treatments

**Figure 6:**
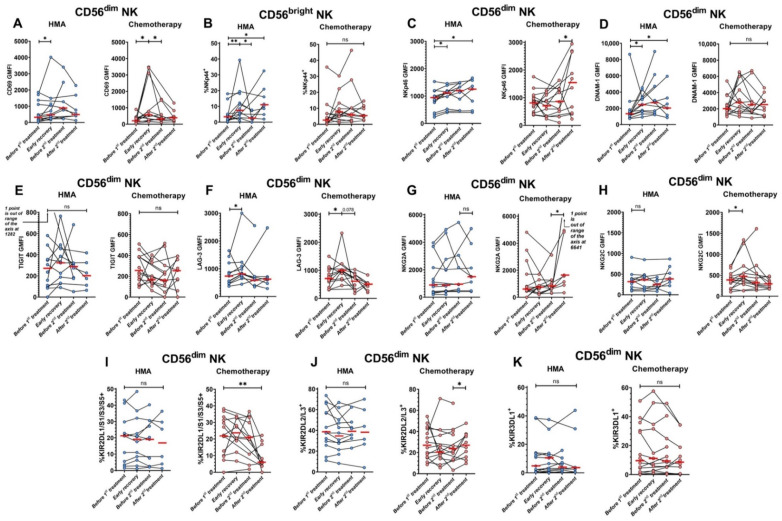
Expression levels of various NK cell surface receptors before and after HMA/venetoclax and cytotoxic therapies. Blue circles are HMA/venetoclax (left panels), pink circles are chemotherapy (right panels), and red bars represent medians. Data are from patients with >1 sample collected. (**A**) CD69 expression on mature CD56^dim^ NK cells. (**B**) Percent of NKp44^+^ immature CD56^bright^ NK cells. (**C**) NKp46 expression on mature CD56^dim^ NK cells. (**D**) DNAM-1 expression on mature CD56^dim^ NK cells. (**E**) TIGIT expression on mature CD56^dim^ NK cells. (**F**) LAG-3 expression on mature CD56^dim^ NK cells. (**G**) NKG2A expression on mature CD56^dim^ NK cells. (**H**) NKG2C expression on mature CD56^dim^ NK cells. (**I**) Change in % CD56^dim^ NK cells expressing KIR2DL1/S1/S3/S5. (**J**) Change in % CD56^dim^ NK cells expressing KIR2DL2/L3. (**K**) Change in % CD56^dim^ NK cells expressing KIR3DL1. Wilcoxon signed-rank test was used for statistical analysis. ns—*p* > 0.05; *—*p* < 0.05; **—*p* < 0.01. A p-value is specified in cases when it is close to 0.05.

We also focused on monitoring a wide variety of NK cell biomarkers in the antibody panel. Expression of the activation marker CD69 increased on mature CD56^dim^ NK cells after either initial HMA/venetoclax or IC treatments (Figure 6A). Upon assessing natural cytotoxicity receptors (NCRs; NKp30, NKp44, NKp46 [28]), we found that the percentage of NKp44^+^ immature CD56^bright^ NK cells increased after the initial HMA/venetoclax treatment, but not IC (Figure 6B). Expression of NKp46 was upregulated on mature NK cells after initial HMA/venetoclax treatment and increased for both groups through the second treatment period (Figure 6C), whereas expression of NKp30 was unchanged by either treatment.

Mature NK cells from patients treated with HMA/venetoclax also showed enhanced expression of the activating receptor DNAM-1 in the early recovery period (Figure 6D), while expression of the inhibitory checkpoint receptor TIGIT, which shares the same ligands as DNAM-1, was unchanged in both groups of patients (Figure 6E). Expression of the inhibitory checkpoint receptor LAG-3 increased on these mature NK cells in both treatment groups after initial therapy (Figure 6F).

We also observed significant changes to NK cell phenotype that were specifically restricted to the IC-treated group. IC induced a higher expression of the inhibitory receptor NKG2A on mature NK cells after consolidation treatment (Figure 6G). The treatment also increased expression of the related activating receptor NKG2C after induction therapy (Figure 6H).

KIRs are stochastically expressed on individual CD56^dim^ NK cells, and their expression is strongly modulated by DNA methylation within promoters [29]. The percentages of CD56^dim^ NK cells expressing particular members of the KIR family can differ between individuals but are consistent within an individual over time [20,30]. In vitro treatment with HMA significantly enhances KIR expression through demethylation [31]. However, we found no statistically significant change in the percentage of CD56^dim^ NK cells expressing various KIRs in the blood of patients throughout the course of HMA/venetoclax therapy (Figure 6I–K). In contrast, significant and markedly wider shifts in percentages of NK cells expressing various KIRs on CD56^dim^ NK cells were observed in the IC-treated group across the four timepoints, presumably due to significant depletion and regeneration of the overall NK cell population. The distribution of KIR expression through therapy course for individual patients is also shown in Supplementary Figure S1F.

### 3.9. Venetoclax-Resistant Patients Exhibit a Distinct T Cell Phenotype

**Figure 7:**
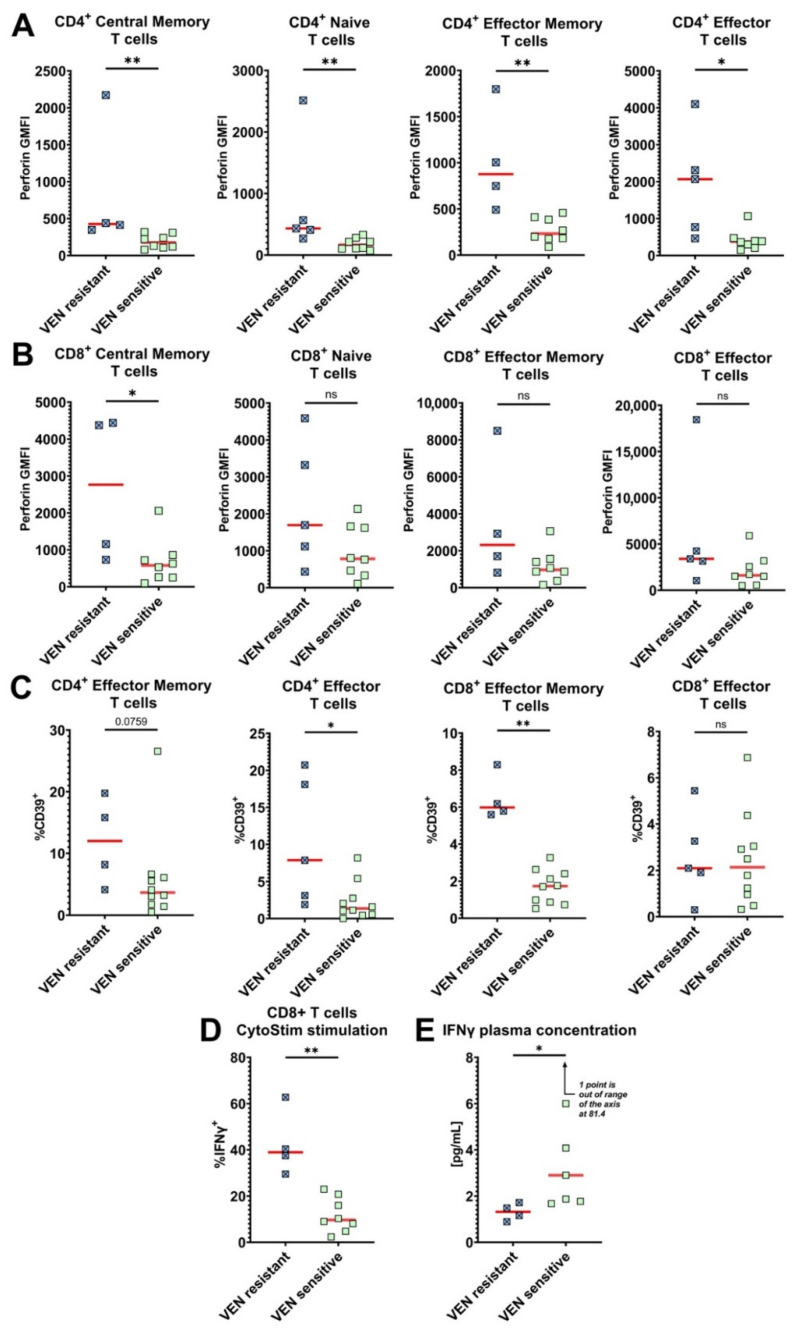
Distinct characteristics of T cells from venetoclax-resistant patients. The 15 patients treated with the combination of HMA/venetoclax were compared for different biomarker expression profiles. Five patients were venetoclax-resistant (VEN resistant), and ten were venetoclax-sensitive (VEN sensitive). One venetoclax-resistant patient (#14) lacked memory T cell subpopulations, so plots of effector memory and central memory T cells have four data points. (**A**) Intracellular GMFI expression of perforin by CD4^+^ T cells. (**B**) Intracellular GMFI expression of perforin by CD8^+^ T cells. Two patients in panels A and B were not assayed due to technical error. (**C**) Proportion of effector memory and effector T cells expressing CD39. (**D**) % IFN-γ-producing CD8^+^ T cells after in vitro stimulation with CytoStim. Three patients were not assayed due to low PBMC availability. (**E**) The level of IFN-γ in plasma from venetoclax-resistant and venetoclax-sensitive AML patients. MesoScale-based analysis was performed for 11 HMA/venetoclax-treated patients. Red bars represent medians. Mann–Whitney nonparametric U test was used for statistical analysis. ns—*p* > 0.05; *—*p* < 0.05; **—*p* < 0.01. A *p*-value is specified when it is close to 0.05.

Venetoclax therapeutic resistance is an important clinical problem that significantly lowers the efficacy of HMA/venetoclax treatment [32]. Evaluation of venetoclax resistance was performed based on the results of bone marrow biopsy after the first two cycles of treatment. Venetoclax resistance was observed in five of fifteen patients treated with HMA/venetoclax. Therefore, we searched for immune phenotypes and functions that may predict resistance by comparing biomarker expression in pre-treatment samples from venetoclax-resistant vs. responsive patients. First, pre-treatment intracellular perforin expression was significantly higher in all CD4^+^ T cell subpopulations of venetoclax-resistant patients compared to those that responded (Figure 7A), suggesting an enhanced cytolytic potential of the CD4^+^ T cells [33] in the resistant patients. Baseline perforin expression was also higher in the CD8^+^ T cell subpopulations of some venetoclax-resistant patients but only reached the threshold of statistical significance in the central memory subset (Figure 7B). Within the venetoclax-resistant patients, CD4^+^ effector and CD8^+^ effector memory T cells also expressed higher levels of the exhaustion marker CD39, and the same trend was observed for CD4^+^ effector memory T cells (Figure 7C). When we stimulated T cells with CytoStim, a significantly higher proportion of CD8^+^ T cells produced IFN-γ from venetoclax-resistant patients (Figure 7D). In stark contrast, we observed a reverse relationship between the increased percentage of CD8^+^ T cells capable of producing IFN-γ in vitro and the lower level of IFN-γ in patient plasma (Figure 7E). These findings identify new phenotypical features of T cells in baseline peripheral blood of patients that subsequently exhibit venetoclax-resistance, which could be potential biomarkers to identify resistant patients prior to the onset of initial therapy.

## 4. Discussion

Two current standard-of-care treatments for AML are cytotoxic IC and HMAs, with the latter most often administered to older patients in combination with the Bcl-2 inhibitor venetoclax. Our primary goal was to assess the impacts of these frontline therapies on lymphocyte phenotypes and functions to consider whether a follow-up course of immunotherapy could be considered. In the course of our studies, we also compared the immune cell phenotypes and functions of these previously untreated AML patients with HDs. Finally, we explored specific features of lymphocytes from venetoclax-resistant patients.

Our observations of immune status in the untreated AML patients were consistent with increased lymphocyte exhaustion, as previously reported [13,14,15], including enhanced PD-1 immune checkpoint expression [34], decreased DNAM-1 activating receptor expression on NK cells [35], and potent upregulation of the exhaustion and immunosuppressive marker CD39 on B, NK, and CD4^+^ T lymphocytes. Our findings revealed a shift to more mature lymphocyte differentiation in patients with decreased CD27 expression by T cells and increased CD57 expression on NK cells. We showed a reduced capacity to stimulate IFN-γ production in vitro by NK cells, but not T cells, from AML patients. Finally, AML patients had a higher prevalence of CD57^+^NKG2C^+^ adaptive NK cells than HDs, and many of these patients were surprisingly HCMV-seronegative. In contrast, adaptive NK cells in the peripheral blood of otherwise healthy donors are classically associated with HCMV infection [36,37].

HMA treatment is considered to be milder than IC and causes toxic effects only in high doses [38,39]. However, the overall toxicity of HMA/venetoclax combination therapy toward lymphocytes did not differ from IC in our study. Severe B lymphopenia was especially pronounced. NK cells were the only lymphocytes showing slight recovery following IC at the final timepoint, with notably increased expansion of immature CD56^bright^ NK cells. These results suggest subsequent immunotherapeutic approaches targeting NK cells could be more beneficial earlier than therapies targeting T or B cells, but significant lymphocyte loss was still evident two to three months after original induction therapy. The shift to a greater percentage of effector memory T cells could be the result of increased differentiation in this direction or lower sensitivity of this subpopulation to the toxic effects of HMA therapy. It should also be noted that the HMA/venetoclax-treated patients were about 13 years older, on average, which may have impacted sensitivity to their course of treatment.

Treatment with HMA/venetoclax also strongly inhibited in vitro IFN-γ production by CD8^+^ T cells and increased perforin expression in mature NK cells. We stimulated T cells with CytoStim (Miltenyi), which crosslinks the TCR, suggesting that TCR signaling is impaired in the CD8^+^ T cells of AML patients. Despite the suppressed IFN-γ response by CD8^+^ T cells, IFN-γ was paradoxically increased in the plasma of the same AML patients. Although the source of circulating IFN-γ is unknown, it could be inducing PD-L1 expression to drive CD8^+^ T cells into an exhausted state [40]. The increased perforin expression could be due to HMA exposure since in vitro activation of human CD8^+^ T cells in the presence of decitabine has been shown to upregulate perforin and granzyme B [41].

We also found decreased surface expression of immune checkpoint receptors PD-1 and 2B4 on CD4^+^ T cells after HMA/venetoclax, but not IC, therapy. In contrast, some studies suggested that HMAs induce PD-1 expression, which prompted several clinical trials of combination HMA + PD-1-blocking therapies [42,43,44,45]. However, most of these studies measured increased *PDCD1* mRNA levels in cell lines or blast cells, usually in vitro. Orskov et al. [43] showed that some, but not all, 5-azacytidine-treated patients exhibited *PDCD1* promoter demethylation in T cells, and only two of these patients were studied to show parallel modest increases in PD-1 protein expression on T cells. However, we did observe increased CTLA-4 expression on Treg cells after treatment with HMA/venetoclax, consistent with a previous report of increased CTLA-4 mRNA expression in HMA-treated patients [44]. In addition, Ki67 was increased in immunosuppressive Tregs after the initial HMA/venetoclax treatment, indicating their proliferation, which already constituted a more prevalent fraction of total CD4^+^ T cells prior to treatment of these patients.

In searching the literature, we noted that increased expression of the B lymphocyte-induced maturation protein 1 (Blimp-1) transcription factor could similarly regulate many of the phenotypic changes induced by HMA/venetoclax in our AML patients. In addition, Blimp-1 expression can be upregulated by in vitro treatment with HMAs [46,47]. For example, Blimp-1 was reported to be overexpressed in CD62L^–^ effector and effector memory T cells and was required for effector T cell differentiation [48,49], and we observed an increased percentage of effector memory T cells in treated patients. Blimp-1 also inhibits IFN-γ secretion by T cells [50] and increases their perforin and granzyme B expression [51,52,53]. While there are contradicting reports on the impact of Blimp-1 on PD-1 expression, Zhu et al. have demonstrated that a high expression of Blimp-1 in CD4^+^ T cells is associated with a higher frequency of blasts in AML [54,55]. Finally, Blimp-1 deficiency impairs proliferation of Treg cells [56]. Therefore, we hypothesized that demethylation of the *Blimp-1* promoter by HMAs may contribute to the phenotypic and functional changes we observed. To test this hypothesis, we were able to stain PBMCs from three of the AML patients for Blimp-1 protein before and after treatment and observed strong upregulation in B, T, and NK cells after HMA/venetoclax therapy (Supplementary Figure S1G,H). The analysis of more patients and more mechanistic studies are required, but our preliminary results suggest that HMA-induced demethylation of the Blimp-1 promoter may contribute to some of the immune impacts observed in treated patients.

Although NK cell expression of the CD69 activation marker was increased in response to either therapy, only HMA/venetoclax treatment upregulated NK cell expression of activating DNAM-1 receptor, which was reduced in untreated AML patients compared to HDs. Moreover, HMA/venetoclax potentiated the expression of NKp44 and NKp46 activating receptors on NK cells. In contrast, IC increased levels of inhibitory NKG2A, thereby shifting the balance toward inhibitory signaling. Notably, we were surprised by a stable KIR expression on NK cells in HMA/venetoclax-treated patients, despite several reports that in vitro treatment with HMAs increases KIR expression [31].

Lastly, we found several unique T cell signatures that were enriched in pre-treatment blood of patients that ultimately exhibited venetoclax resistance. T cells from the venetoclax-resistant patients exhibited a higher baseline perforin expression that was most pronounced in CD4^+^ cells, enhanced CD39 expression, and more robust production of IFN-γ when stimulated in vitro. Although the increased perforin in CD4^+^ T cells was unexpected, recent work has shown the importance of anti-tumor responses by cytolytic CD4^+^ T cells [33]. Despite the enhanced capacity to produce IFN-γ, however, venetoclax-resistant patients had lower concentrations of IFN-γ in plasma at the time of initial diagnosis.

## 5. Conclusions

Our data shed light on the immune status of AML patients, provide new insights on the effects of HMA/venetoclax and IC therapies on their lymphocytes, and identify pre-treatment immune biomarkers that may predict venetoclax-resistant patients. Key findings include: (1) Untreated AML patients had numerous signs of lymphocyte exhaustion, particularly in CD4^+^ T and NK cells, as well as a greater incidence of adaptive NK cells that was independent of HCMV serum titer. (2) Both HMA/venetoclax and IC therapies dramatically reduced lymphocytes for up to three months, with only NK cells showing modest signs of recovery in the final blood samples. (3) HMA/venetoclax was more impactful than IC on lymphocyte phenotype and function, including reducing IFN-γ production upon stimulation of CD8^+^ T cells, increasing expression of perforin, CD69, and several receptors in NK cells, decreasing PD-1 and 2B4 expression on CD4^+^ T cells, and increasing proliferation of and CTLA-4 expression by Tregs. (4) Finally, AML patients with higher pre-treatment levels of perforin in T cells, higher in vitro IFN-γ response by CD8^+^ T cells, and lower plasma levels of IFN-γ went on to exhibit venetoclax resistance.

## Data Availability

The raw flow cytometry data collected and analyzed during the study are available from the corresponding author on reasonable request.

## Supplementary Materials

The following supporting information can be downloaded at: https://www.mdpi.com/article/10.3390/cancers14143352/s1, Figure S1: Additional characteristics of AML patients; Figure S2: General scheme of the gating strategy used in the study; Figure S3: Representative staining of various biomarkers; Figure S4: Representative staining of various biomarkers on NK cells; Figure S5: Representative staining of adaptive NK cells; Table S1: Patient characteristics; Table S2: Antibody panel.

## References

[1] Ngai L.L., Kelder A., Janssen J.J.W.M., Ossenkoppele G.J., Cloos J. (2021). MRD tailored therapy in AML: What we have learned so far. Front. Oncol..

[2] Kantarjian H., Kadia T., DiNardo C., Daver N., Borthakur G., Jabbour E., Garcia-Manero G., Konopleva M., Ravandi F. (2021). Acute myeloid leukemia: Current progress and future directions. Blood Cancer J..

[3] Roberts M.D., Langston A.A., Heffner L.T. (2019). Acute myeloid leukemia in young adults: Does everyone need a transplant?. J. Oncol. Pract..

[4] Schiffer C.A. (2014). Optimal dose and schedule of consolidation in AML: Is there a standard?. Best Pract. Res. Clin. Haematol..

[5] Lancet J.E., Uy G.L., Cortes J.E., Newell L.F., Lin T.L., Ritchie E.K., Stuart R.K., Strickland S.A., Hogge D., Solomon S.R. (2018). CPX-351 (cytarabine and daunorubicin) liposome for injection versus conventional cytarabine plus daunorubicin in older patients with newly diagnosed secondary acute myeloid leukemia. J. Clin. Oncol..

[6] Stanchina M., Soong D., Zheng-Lin B., Watts J.M., Taylor J. (2020). Advances in acute myeloid leukemia: Recently approved therapies and drugs in development. Cancers.

[7] Baylin S.B. (2005). DNA methylation and gene silencing in cancer. Nat. Clin. Pract. Oncol..

[8] Talati C., Dhulipala V.C., Extermann M.T., Al Ali N., Kim J., Komrokji R., Sweet K., Kuykendall A., Sehovic M., Reljic T. (2020). Comparisons of commonly used front-line regimens on survival outcomes in patients aged 70 years and older with acute myeloid leukemia. Haematologica.

[9] Vachhani P., Al Yacoub R., Miller A., Zhang F., Cronin T.L., Ontiveros E.P., Thompson J.E., Griffiths E.A., Wang E.S. (2018). Intensive chemotherapy vs. hypomethylating agents in older adults with newly diagnosed high-risk acute myeloid leukemia: A single center experience. Leuk. Res..

[10] Santini V., Ossenkoppele G.J. (2019). Hypomethylating agents in the treatment of acute myeloid leukemia: A guide to optimal use. Crit. Rev. Oncol. Hematol..

[11] Samra B., Konopleva M., Isidori A., Daver N., Dinardo C. (2020). Venetoclax-based combinations in acute myeloid leukemia: Current evidence and future directions. Front. Oncol..

[12] Daver N., Wei A.H., Pollyea D.A., Fathi A.T., Vyas P., DiNardo C.D. (2020). New directions for emerging therapies in acute myeloid leukemia: The next chapter. Blood Cancer J..

[13] Knaus H.A., Berglund S., Hackl H., Blackford A.L., Zeidner J., Montiel-Esparza R., Mukhopadhyay R., Vanura K., Blazar B.R., Karp J.E. (2018). Signatures of CD8^+^ T cell dysfunction in AML patients and their reversibility with response to chemotherapy. JCI Insight.

[14] Li Z., Philip M., Ferrell P.B. (2020). Alterations of T-cell-mediated immunity in acute myeloid leukemia. Oncogene.

[15] Swatler J., Turos-Korgul L., Kozlowska E., Piwocka K. (2021). Immunosuppressive cell subsets and factors in myeloid leukemias. Cancers.

[16] Aldarouish M., Su X., Qiao J., Gao C., Chen Y., Dai A., Zhang T., Shu Y., Wang C. (2019). Immunomodulatory effects of chemotherapy on blood lymphocytes and survival of patients with advanced non-small cell lung cancer. Int. J. Immunopathol. Pharmacol..

[17] Galluzzi L., Buqué A., Kepp O., Zitvogel L., Kroemer G. (2015). Immunological effects of conventional chemotherapy and targeted anticancer agents. Cancer Cell.

[18] Waidhauser J., Schuh A., Trepel M., Schmälter A.-K., Rank A. (2020). Chemotherapy markedly reduces B cells but not T cells and NK cells in patients with cancer. Cancer Immunol. Immunother..

[19] Kanakry C.G., Hess A.D., Gocke C.D., Thoburn C., Kos F., Meyer C., Briel J., Luznik L., Smith B.D., Levitsky H. (2011). Early lymphocyte recovery after intensive timed sequential chemotherapy for acute myelogenous leukemia: Peripheral oligoclonal expansion of regulatory T cells. Blood.

[20] MacFarlane A.W., Jillab M., Smith M.R., Alpaugh R.K., Cole M.E., Litwin S., Millenson M.M., Al-Saleem T., Cohen A.D., Campbell K.S. (2017). NK cell dysfunction in chronic lymphocytic leukemia is associated with loss of the mature cells expressing inhibitory killer cell Ig-like receptors. OncoImmunology.

[21] Balança C.-C., Salvioni A., Scarlata C.-M., Michelas M., Martinez-Gomez C., Gomez-Roca C., Sarradin V., Tosolini M., Valle C., Pont F. (2021). PD-1 blockade restores helper activity of tumor-infiltrating, exhausted PD-1^hi^CD39^+^ CD4 T cells. JCI Insight.

[22] Brauneck F., Seubert E., Wellbrock J., Wiesch J.S.Z., Duan Y., Magnus T., Bokemeyer C., Koch-Nolte F., Menzel S., Fiedler W. (2021). Combined blockade of TIGIT and CD39 or A2AR enhances NK-92 cell-mediated cytotoxicity in AML. Int. J. Mol. Sci..

[23] Gupta P.K., Godec J., Wolski D., Adland E., Yates K., Pauken K.E., Cosgrove C., Ledderose C., Junger W.G., Robson S.C. (2015). CD39 expression identifies terminally exhausted CD8^+^ T cells. PLoS Pathog..

[24] Figueiró F., Muller L., Funk S., Jackson E., Battastini A., Whiteside T. (2016). Phenotypic and functional characteristics of CD39^high^human regulatory B cells (Breg). OncoImmunology.

[25] Schlums H., Cichocki F., Tesi B., Theorell J., Beziat V., Holmes T.D., Han H., Chiang S.C., Foley B., Mattsson K. (2015). Cytomegalovirus infection drives adaptive epigenetic diversification of NK cells with altered signaling and effector function. Immunity.

[26] Bjorkstrom N.K., Riese P., Heuts F., Andersson S., Fauriat C., Ivarsson M.A., Bjorklund A.T., Flodstrom-Tullberg M., Michaelsson J., Rottenberg M.E. (2010). Expression patterns of NKG2A, KIR, and CD57 define a process of CD56^dim^ NK-cell differentiation uncoupled from NK-cell education. Blood.

[27] Rueff J., Medinger M., Heim D., Passweg J., Stern M. (2014). Lymphocyte subset recovery and outcome after autologous hematopoietic stem cell transplantation for plasma cell myeloma. Biol. Blood Marrow Transplant..

[28] Pazina T., Shemesh A., Brusilovsky M., Porgador A., Campbell K.S. (2017). Regulation of the functions of natural cytotoxicity receptors by interactions with diverse ligands and alterations in splice variant expression. Front. Immunol..

[29] Béziat V., Hilton H., Norman P., Traherne J.A. (2016). Deciphering the killer-cell immunoglobulin-like receptor system at super-resolution for natural killer and T-cell biology. Immunology.

[30] Campbell K.S., Hasegawa J. (2013). Natural killer cell biology: An update and future directions. J. Allergy Clin. Immunol..

[31] Lindblad K.E., Goswami M., Hourigan C.S., Oetjen K.A. (2017). Immunological effects of hypomethylating agents. Expert Rev. Hematol..

[32] Saliba A.N., John A.J., Kaufmann S.H. (2021). Resistance to venetoclax and hypomethylating agents in acute myeloid leukemia. Cancer Drug Resist..

[33] Oh D.Y., Kwek S.S., Raju S.S., Li T., McCarthy E., Chow E., Aran D., Ilano A., Pai C.-C.S., Rancan C. (2020). Intratumoral CD4^+^ T cells mediate anti-tumor cytotoxicity in human bladder cancer. Cell.

[34] Xu L., Liu L., Yao D., Zeng X., Zhang Y., Lai J., Zhong J., Zha X., Zheng R., Lu Y. (2021). PD-1 and TIGIT are highly co-expressed on CD8^+^ T cells in AML patient bone marrow. Front. Oncol..

[35] Bi J., Tian Z. (2017). NK cell exhaustion. Front. Immunol..

[36] Cichocki F., Cooley S., Davis Z., DeFor T.E., Schlums H., Zhang B., Brunstein C.G., Blazar B.R., Wagner J.E., Diamond D. (2015). CD56^dim^CD57^+^NKG2C^+^ NK cell expansion is associated with reduced leukemia relapse after reduced intensity HCT. Leukemia.

[37] Merino A., Zhang B., Dougherty P., Luo X., Wang J., Blazar B.R., Miller J.S., Cichocki F. (2019). Chronic stimulation drives human NK cell dysfunction and epigenetic reprograming. J. Clin. Investig..

[38] Al-Ali H.K., Jaekel N., Niederwieser D. (2014). The role of hypomethylating agents in the treatment of elderly patients with AML. J. Geriatr. Oncol..

[39] Stomper J., Rotondo J.C., Greve G., Lübbert M. (2021). Hypomethylating agents (HMA) for the treatment of acute myeloid leukemia and myelodysplastic syndromes: Mechanisms of resistance and novel HMA-based therapies. Leukemia.

[40] Benci J.L., Johnson L.R., Choa R., Xu Y., Qiu J., Zhou Z., Xu B., Ye D., Nathanson K.L., June C.H. (2019). Opposing functions of interferon coordinate adaptive and innate immune responses to cancer immune checkpoint blockade. Cell.

[41] Yau H.L., Bell E., Ettayebi I., de Almeida F.C., Boukhaled G.M., Shen S.Y., Allard D., Morancho B., Marhon S.A., Ishak C.A. (2021). DNA hypomethylating agents increase activation and cytolytic activity of CD8^+^ T cells. Mol. Cell.

[42] Daver N., Garcia-Manero G., Basu S., Boddu P.C., Alfayez M., Cortes J.E., Konopleva M., Ravandi-Kashani F., Jabbour E., Kadia T. (2019). Efficacy, safety, and biomarkers of response to azacitidine and nivolumab in relapsed/refractory acute myeloid leukemia: A nonrandomized, open-label, phase II study. Cancer Discov..

[43] Ørskov A.D., Treppendahl M.B., Skovbo A., Holm M.S., Friis L.S., Hokland M., Grønbæk K. (2015). Hypomethylation and up-regulation of *PD-1* in T cells by azacytidine in MDS/AML patients: A rationale for combined targeting of PD-1 and DNA methylation. Oncotarget.

[44] Yang H., Bueso-Ramos C., Dinardo C., Estecio M.R., Davanlou M., Geng Q.-R., Fang Z., Nguyen M., Pierce S., Wei Y. (2014). Expression of PD-L1, PD-L2, PD-1 and CTLA4 in myelodysplastic syndromes is enhanced by treatment with hypomethylating agents. Leukemia.

[45] Zhang M., Xiao X.Q., Jiang Y.F., Liang Y.S., Peng M.Y., Xu Y., Gong G.Z. (2011). DNA demethylation in PD-1 gene promoter induced by 5-azacytidine activates PD-1 expression on Molt-4 cells. Cell. Immunol..

[46] Zhang T., Ma J., Nie K., Yan J., Liu Y., Bacchi C.E., Queiroga E.M., Gualco G., Sample J.T., Orazi A. (2014). Hypermethylation of the tumor suppressor gene PRDM1/Blimp-1 supports a pathogenetic role in EBV-positive Burkitt lymphoma. Blood Cancer J..

[47] Zhang Z., Liang L., Li D., Nong L., Liu J., Qu L., Zheng Y., Zhang B., Li T. (2017). Hypermethylation of PRDM1/Blimp-1 promoter in extranodal NK/T-cell lymphoma, nasal type: An evidence of predominant role in its downregulation. Hematol. Oncol..

[48] Fu S.-H., Yeh L.-T., Chu C.-C., Yen B.L.-J., Sytwu H.-K. (2017). New insights into Blimp-1 in T lymphocytes: A divergent regulator of cell destiny and effector function. J. Biomed. Sci..

[49] Kallies A., Xin A., Belz G.T., Nutt S.L. (2009). Blimp-1 transcription factor is required for the differentiation of effector CD8^+^ T cells and memory responses. Immunity.

[50] Cimmino L., Martins G.A., Liao J., Magnusdottir E., Grunig G., Perez R.K., Calame K.L. (2008). Blimp-1 attenuates Th1 differentiation by repression of *ifng*, *tbx21*, and *bcl6* gene expression. J. Immunol..

[51] Hua L., Yao S., Pham D., Jiang L., Wright J., Sawant D., Dent A.L., Braciale T.J., Kaplan M.H., Sun J. (2013). Cytokine-dependent induction of CD4^+^ T cells with cytotoxic potential during influenza virus infection. J. Virol..

[52] Rutishauser R.L., Martins G.A., Kalachikov S., Chandele A., Parish I.A., Meffre E., Jacob J., Calame K., Kaech S.M. (2009). Transcriptional repressor Blimp-1 promotes CD8^+^ T cell terminal differentiation and represses the acquisition of central memory T cell properties. Immunity.

[53] Shin H., Blackburn S.D., Intlekofer A.M., Kao C., Angelosanto J.M., Reiner S.L., Wherry E.J. (2009). A role for the transcriptional repressor Blimp-1 in CD8^+^ T cell exhaustion during chronic viral infection. Immunity.

[54] Lu P., Youngblood B., Austin J.W., Mohammed A.U.R., Butler R., Ahmed R., Boss J.M. (2014). Blimp-1 represses CD8 T cell expression of PD-1 using a feed-forward transcriptional circuit during acute viral infection. J. Exp. Med..

[55] Zhu L., Kong Y., Zhang J., Claxton D.F., Ehmann W.C., Rybka W.B., Palmisiano N.D., Wang M., Jia B., Bayerl M. (2017). Blimp-1 impairs T cell function via upregulation of TIGIT and PD-1 in patients with acute myeloid leukemia. J. Hematol. Oncol..

[56] Lin M.-H., Yeh L.-T., Chen S.-J., Chiou H.-Y.C., Chu C.-C., Yen L.B., Lin K.-I., Chang D.-M., Sytwu H.-K. (2014). T cell-specific BLIMP-1 deficiency exacerbates experimental autoimmune encephalomyelitis in nonobese diabetic mice by increasing Th1 and Th17 cells. Clin. Immunol..

