# Supplementary material for: Lymphocyte Exhaustion in AML Patients and Impacts of HMA/Venetoclax or Intensive Chemotherapy on Their Biology
Source: Cancers (Basel). 2022 Jul 10;14(14):3352. doi: 10.3390/cancers14143352 (PMC9320805; doi:10.3390/cancers14143352)
Supplement: Supplementary file 1 [file cancers-14-03352-s001.zip › cancers-1730050-supplementary.pdf]

cancers

Supplementary Material: Lymphocyte Exhaustion in AML Patients and Impacts of HMA/Venetoclax or Intensive Chemotherapy on Their Biology

Dmitry Zhigarev, Asya Varshavsky, Alexander W. MacFarlane IV, Prathiba Jayaguru, Laura Barreyro, Marina Khoreva, Essel Dulaimi, Reza Nejati, Christina Drenberg and Kerry S. Campbell

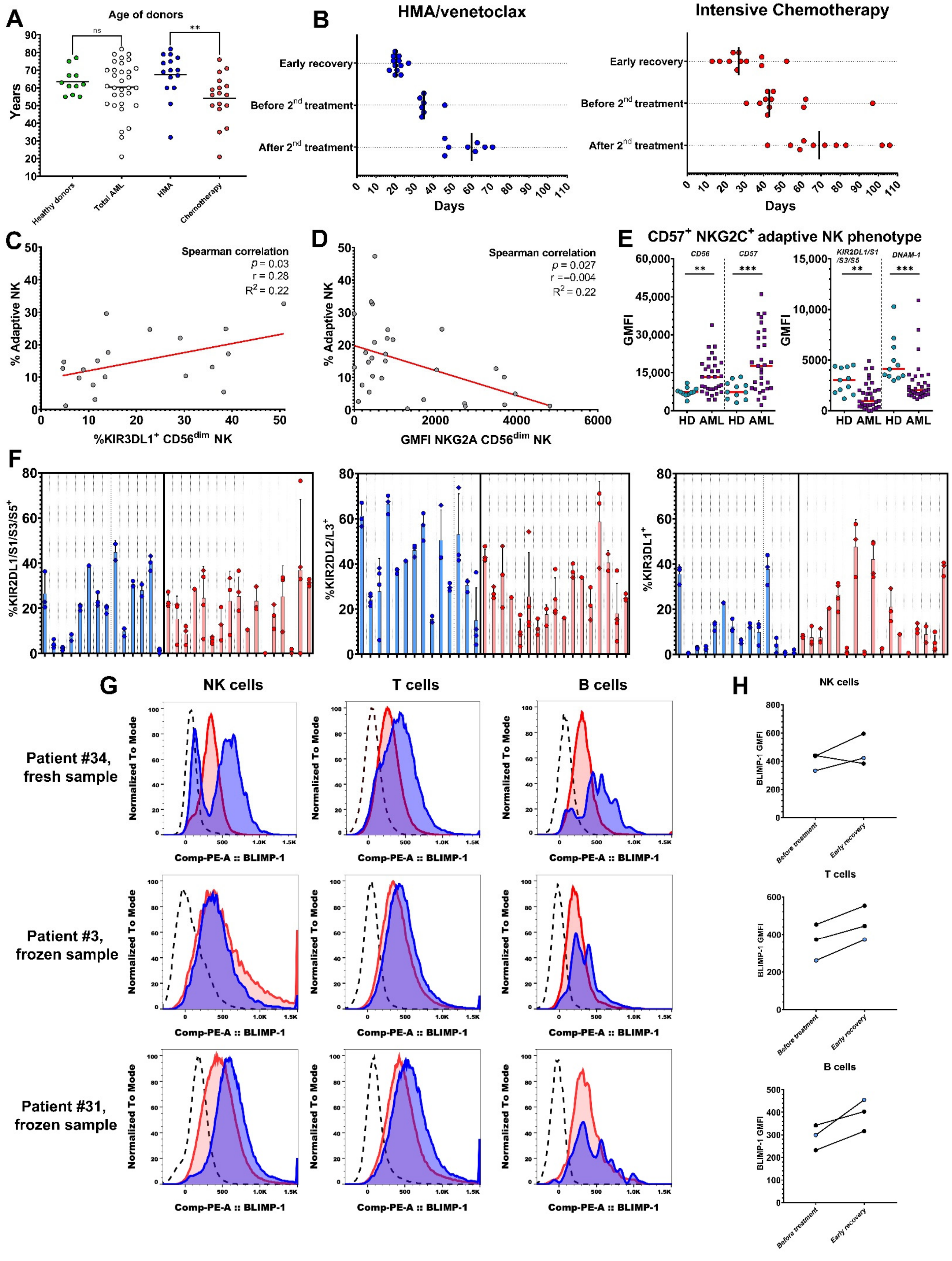
A
Age of donors
ns
**
Years
Healthy donors
Total AML
HMA
Chemotherapy
B
HMA/venetoclax
Intensive Chemotherapy
Early recovery
Before 2nd treatment
After 2nd treatment
Days
C
Spearman correlation
p = 0.03
r = 0.28
R2 = 0.22
% Adaptive NK
%KIR3DL1+ CD56dim NK
D
Spearman correlation
p = 0.027
r = −0.004
R2 = 0.22
% Adaptive NK
GMFI NKG2A CD56dim NK
E
CD57+ NKG2C+ adaptive NK phenotype
CD56
CD57
KIR2DL1/S1/S3/S5
DNAM-1
GMFI
HD AML HD AML
F
%KIR2DL1/S1/S3/S5+
%KIR2DL2/L3+
%KIR3DL1+
G
NK cells
T cells
B cells
Patient #34, fresh sample
Patient #3, frozen sample
Patient #31, frozen sample
Normalized To Mode
Comp-PE-A :: BLIMP-1
H
NK cells
T cells
B cells
BLIMP-1 GMFI
Before treatment
Early recovery

Figure S1. Additional characteristics of AML patients. (**A**) Age of individual donors. Green circles show the HD cohort and open circles are total AML patient cohort. Black lines represent means. (**B**) The timing of blood sampling for AML patients. Blue circles display HMA/venetoclax and red circles show IC treated AML patients. Black lines mark medians. (**C**) Positive Spearman correlation of % adaptive NK cells and % KIR3DL1$^+$ total mature NK cells from untreated AML patients. (**D**) Negative Spearman correlation of % adaptive NK cells and % NKG2A$^+$ mature NK cells from untreated AML patients. (**E**) GMFI expression levels of CD56, CD57, KIR2DL1/S1/S3/S5 and DNAM-1 on adaptive NK cells in HD and patients. Red lines mark medians (**F**) Changes in % CD56^{dim} NK cells expressing KIR2DL1/S1/S3/S5, KIR2DL2/L3, or KIR3DL1 during HMA/venetoclax (blue columns) and IC (pink columns). Each column shows mean values and SD for a particular patient with each circle a measure at one of the three post-treatment time points, and pretreatment samples marked as diamonds. (**G**) Blimp-1 expression on lymphocytes from three AML patients before and after initial HMA treatment. Black dashed lines are isotype control, shaded lines represent intensity of BLIMP-1 staining before (red) and after (blue) HMA treatment. (**H**) Blimp-1 expression changes on NK, T, and B cells from three AML patients before and after initial HMA treatment. Black dots represent samples, previously frozen in FBS with 10% DMSO and stored in liquid nitrogen. In (**A**) and (**E**): Mann-Whitney nonparametric U test was used for statistical analysis. ns—$p > 0.05$; **—$p < 0.01$; ***—$p < 0.001$. In (**C**) and (**D**): Spearman's rank correlation coefficient (r), and coefficient of determination (R^2) are displayed in correlation plots. A red line is a linear regression line to better visualize the correlation.

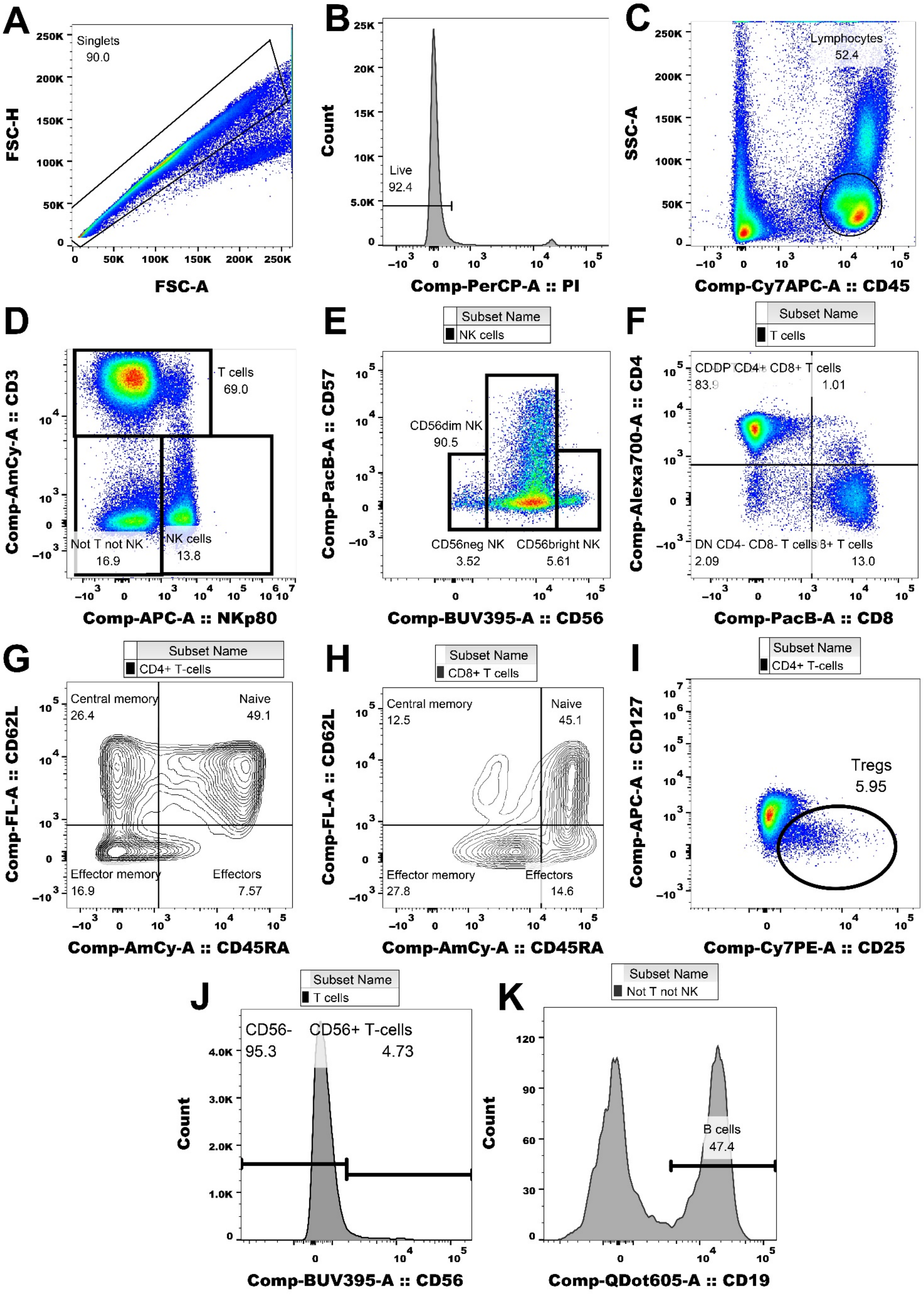

A
Singlets
90.0
FSC-H
FSC-A
B
Live
92.4
Count
Comp-PerCP-A :: PI
C
Lymphocytes
52.4
SSC-A
Comp-Cy7APC-A :: CD45
D
T cells
69.0
Not T not NK
16.9
NK cells
13.8
Comp-AmCy-A :: CD3
Comp-APC-A :: NKp80
E
Subset Name
NK cells
CD56dim NK
90.5
CD56neg NK
3.52
CD56bright NK
5.61
Comp-PacB-A :: CD57
Comp-BUV395-A :: CD56
F
Subset Name
T cells
CD4+ CD8+ T cells
1.01
83.9
DN CD4- CD8- T cells
2.09
13.0
Comp-Alexa700-A :: CD4
Comp-PacB-A :: CD8
G
Subset Name
CD4+ T-cells
Central memory
26.4
Naive
49.1
Effector memory
16.9
Effectors
7.57
Comp-FL-A :: CD62L
Comp-AmCy-A :: CD45RA
H
Subset Name
CD8+ T cells
Central memory
12.5
Naive
45.1
Effector memory
27.8
Effectors
14.6
Comp-FL-A :: CD62L
Comp-AmCy-A :: CD45RA
I
Subset Name
CD4+ T-cells
Tregs
5.95
Comp-APC-A :: CD127
Comp-Cy7PE-A :: CD25
J
Subset Name
T cells
CD56-
95.3
CD56+ T-cells
4.73
Count
Comp-BUV395-A :: CD56
K
Subset Name
Not T not NK
B cells
47.4
Count
Comp-QDot605-A :: CD19

Figure S2. General scheme of the gating strategy used in the study. (**A**) Single cells were selected in a forward scatter area (FSC-A) vs forward scatter height (FSC-H) plot. (**B**) Then, living cells were selected as propidium iodide (PI)-negative or Ghost Red 710-negative (for subsequently fixed and permeabilized samples; Tonbo Bioscience). (**C**) Lymphocytes were identified as $CD45^{+}SSC^{low}$ cells in a CD45 vs side scatter area (SSC-A) dot plot. (**D**) NK cells were defined as $CD3^{-}NKp80^{+}$ lymphocytes (or as $CD3^{-}CD56^{+}$ lymphocytes in tubes with no NKp80 antibodies). T cells were defined as $CD3^{+}$ lymphocytes. The population of $CD3^{-}$ $NKp80^{-}$ lymphocytes was defined as "not T not NK cells". (**E**) Total NK cell population then was divided into $CD56^{dim}$, $CD56^{bright}$, and $CD56^{neg}$ cells in a CD56 vs. CD57 plot. (**F**) $CD4^{+}$, $CD8^{+}$, double-positive and double-negative T cells were separated in CD8 vs. CD4 plot. (**G**) $CD4^{+}$ and (**H**) $CD8^{+}$ T cells were plotted in CD45RA vs. CD62L coordinates and subpopulations of naïve ($CD45RA^{+}$ $CD62L^{+}$), central memory ($CD45RA^{-}$ $CD62L^{+}$), effector memory ($CD45RA^{-}$ $CD62L^{-}$) and effector ($CD45RA^{+}$ $CD62L^{-}$) T cells were selected. (**I**) Additionally, Tregs were defined as $CD25^{high}$ $CD127^{-}$ $CD4^{+}$ T cells, and (**J**) $CD56^{+}$ T cells were selected. (**K**) "Not T not NK cell" population was used for gating B cells as $CD19^{+}$.

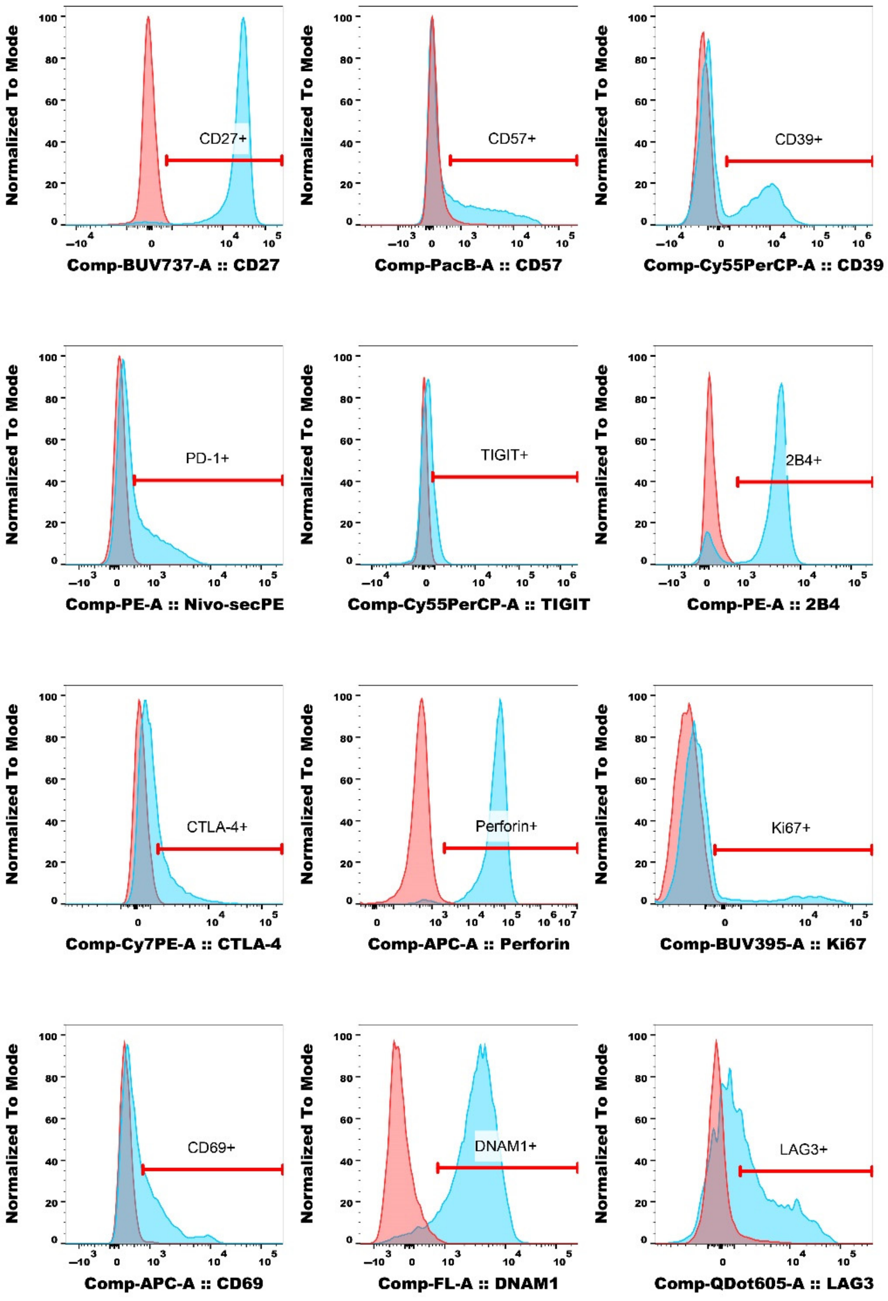

Figure S3. Representative staining of various biomarkers. CD27 (gated on total T cells), CD57 (gated on CD56^{dim} NK cells), CD39 (gated on B cells), PD-1 (gated on CD4^{+} T cells), TIGIT (gated on CD8^{+} central memory T cells), 2B4 (gated on CD8^{+} effector T cells), CTLA-4 (gated on Tregs), Perforin (gated on CD56^{dim} NK cells), Ki67 (gated on Tregs), CD69 (gated on CD8^{+} T cells), DNAM-1 (gated on CD56^{dim} NK cells), and LAG-3 (gated on CD56^{+} T cells) were stained with antibodies described in Supplementary Table S2. Red histograms represent IgG controls or non-expressing population staining. Blue histograms show actual staining from a healthy donor who participated in the study. Gates for % positive cells were set where <1% of cells were gated in control histograms.

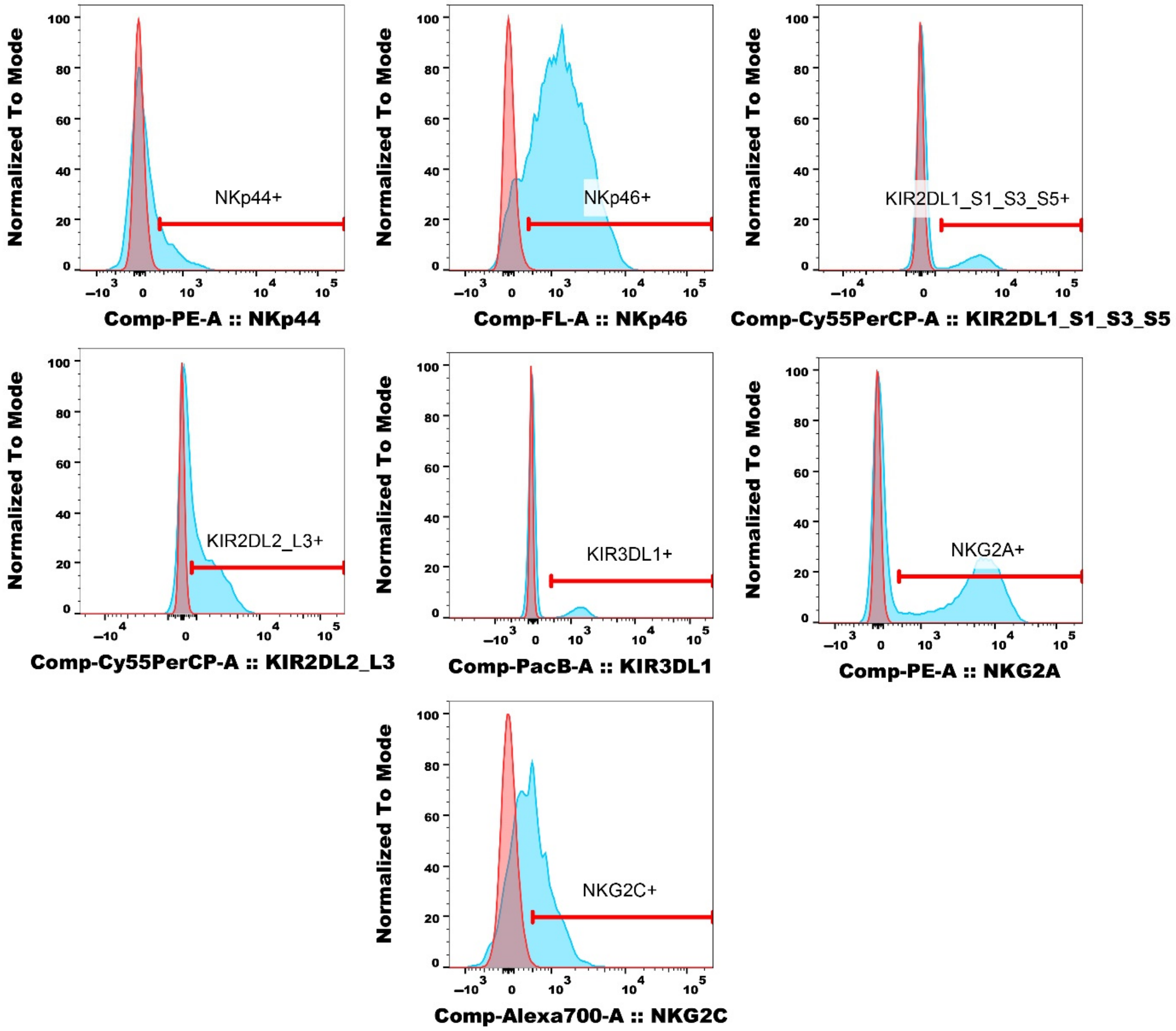

Figure S4. Representative staining of various biomarkers on NK cells. NKp44, NKp46, KIR2DL1/S1/S3/S5, KIR2DL2/L3, KIR3DL1, NKG2A, and NKG2C were stained with antibodies described in Supplementary Table S2. Red histograms represent IgG controls or non-expressing population staining. Blue histograms show actual staining from a healthy donor who participated in the study. Gates for % positive cells were set where <1% of cells were gated in control histograms.

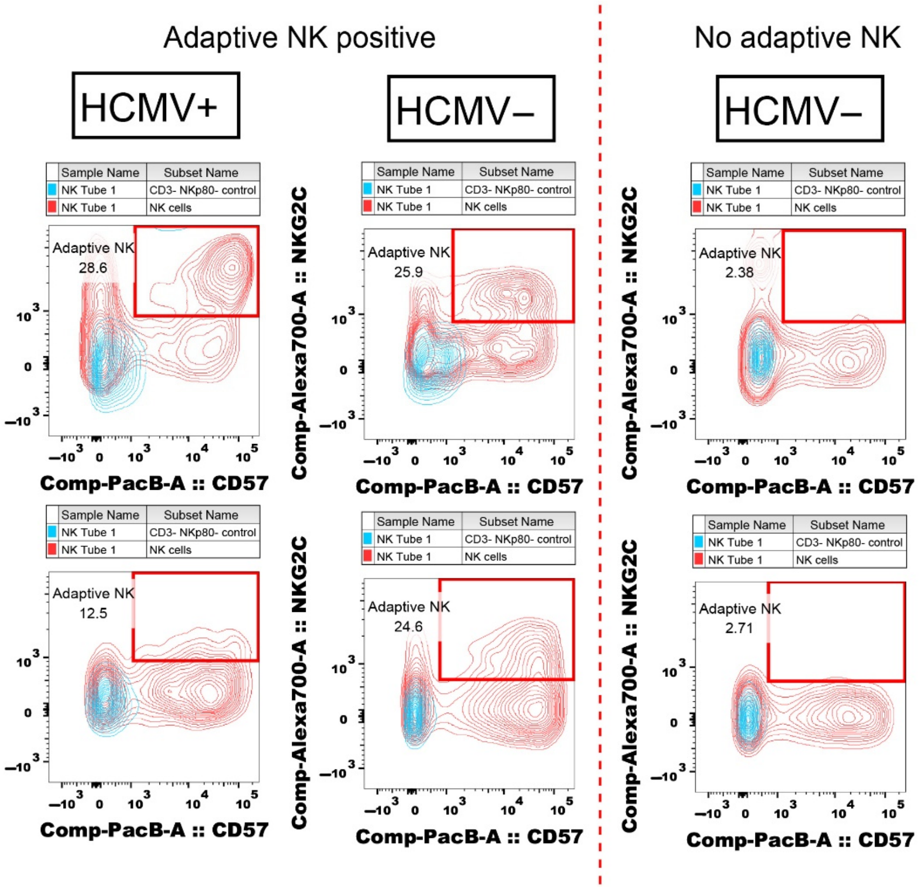

Figure S5. Representative staining of adaptive NK cells. NK cells were gated as $CD3^-NKp80^+$, as in Figures 2A and S2D, and contour 2D plots are shown for staining of NKG2C vs. CD57 to delineate adaptive NK cells as $NKG2C^+$ $CD57^+$ (red boxes) in peripheral blood of six representative untreated AML patients. Superimposed plots are shown for staining of these markers on the NK cells (red), as compared to non-T/non-NK cells ($CD3^-$ $NKp80^-$; blue), as a control. Percentages of $NKG2C^+$ $CD57^+$ adaptive NK cells (within the red boxes) of the total NK cells are indicated. Each column shows two examples of $HCMV^+$ or $HCMV^-$ patients shown to possess (left of dotted line) or lack (right of dotted line) adaptive NK cells. Top panels of patients with adaptive NK cells show bimodal staining for NKG2C, whereas bottom panels show more diffuse NKG2C staining that is clearly above staining of the control cells.

Table S1. Patient characteristics.

Patient No.	Age at Diagnosis	Gender	Cytogenetics and Mutations	Treatment	Outcomes	ELN 2017 Risk	Samples Collected
1	56	Male	45XY, -7[4],46, XY[17]. Mut: EZH1, RUNX1	Vyxeos	CR	poor	4
2	77	Male	46XY Mut: FLT3-ITD high	Decitabine/venetoclax	CRi	poor	4
3	75	Male	45,X,-Y[2]/45,sl,add(6)(p22)[4]/46,sdl,+r[14] Mut: NRAS, TET2, ZRSR2	Decitabine/venetoclax	NR	intermediate	4
4	56	Female	46XX, del5q Mut: DNMT3A, FLT3-TKD	7+3/Midostaurin, FLAG-ida	NR to induction, CRi reinduction	poor	4
5	64	Male	46,XY,inv(16)(p13,1q22)[15]/47,sl,+22[1]/49,sdl,9,+13[2]/46,sl,r(7)(p22q36)[3]46.XY[1] Mut: FLT3-ITD low	7+3/Midostaurin	CR	favorable	4
6	76	Male	46XY, -21q22, Mut: FLT3-ITD low	7+3	NR	intermediate	1
7	79	Female	46XX, del12p Mut: FLT3-ITD low, SRSF2, TET2	Decitabine/venetoclax	CR	intermediate	4
8	48	Female	46XX, Mut: NPM1, IDH2, DNMT3A, NRAS	7+3, HiDAC	CR	favorable	4
9	69	Female	46XX, Mut: NPM1, FLT3-ITD low, TET2	7+3/Midostaurin	CR	favorable	4
10	51	Female	46XX, t(8,21)	7+3/HiDAC	CR	favorable	4
11	32	Female	46XX, Mut: CEBPA, NRAS, WT1	5-azacytidine/venetoclax	CR	favorable	3
12	50	Female	46XX, Mut: FLT3-ITD high	7+3+ midostaurine, HiDAC+midostaurine	CR	poor	4
13	61	Female	46XX, Mut: FLT3-ITD low, NPM1, TET2	7+3+ midostaurine/HiDAC+midostaurine	CR	favorable	4
14	60	Male	44XX, -5,-7,del12p, -18, r(1), -x, del(13)+8, add 10p11, add 16q22, add 20q11 Mut: TP53, TET2	Decitabine/venetoclax	NR	poor	4
15	79	Male	46,XY,t(3;21)(q26;q22),del(7)(q22q36),add(19)(p13.1)[20] , Mut: PTPN11	5-azacytidine/venetoclax	not assessed	poor	1
16	35	Female	46XX, inv (16) Mut: NRAS, WT1	7+3	CR	favorable	1
17	60	Male	46XY, Mut: SRSF2.	7+3, HiDAC	CR	intermediate	4
18	70	Female	50XX, trisomy 8, tetrasomy 11, trisomy 13, del 17p, Mut: TP53, GNAS	Decitabine/venetoclax	CR	poor	3
19	37	Male	47,XY,+11[20], Mut: IDH1	7+3, FLAG-ida	NR	intermediate	1
20	66	Male	40~41,XY,add(3)(q12),der(4;7)(q10;p10),add(5)(q11.2),-6,add(8)(q24.2),-9,-13,-15,der(15;16)(q10;q10),-17,-18,add(22)(q13),+3~4mar[cp11]/46,XY[9] Mut: JAK2, TP53	5-azacytidine/venetoclax	NR	poor	4
21	21	Female	46XX, Mut: CALR, DNMT3A, IDH1, NF1	7+3, HiDAC	NR	intermediate	3
22	51	Male	45~47,XY,-2,del(5)(q22q35),der(9)del(9)(p13)add(9)(q34),del(10)(p11.2),-12,-15,-17,+18,del(18)(q21),der(18)t(2;18)(q13;q21),+r,+2mar[20], Mut: TP53	5-azacytidine/venetoclax	NR	poor	3
23	57	Male	46XY, KMT2A rearrangement, Mut: ASXL1	Vyxeos	CR	poor	3
24	82	Male	46XY, Mut: IDH1, SRSF2, BCOR, DNMT3A	Decitabine/venetoclax	NR	intermediate	3

25	70	Female	42-46,XX,del(5)(q13q33),del(7)(q21.2q36),-13, t(14;21)(q11.2;q22), add(19)(p13.3),+0-1mar[cp18]/46, XX[2]	Decitabine/venetoclax	CR	poor	4
26	69	Male	45-51,XY,t(3;12)(q27;q13),-5,del(6)(q15q21),del(7)(q21),+1-9mar[cp15]/46,X Y[5] , Mut: TP53	5-azacytidine/venetoclax	CR	poor	3
27	59	Male	46XY, Mut: JAK2, WT1, PTPN1	Vyxeos	CR	intermediate	2
28	67	Male	46XY	5-azacytidine/venetoclax	CR	intermediate	3
29	60	Male	46XY, Mut: TP53, SRSF2, TET2	Decitabine/venetoclax	CR	poor	4
30	50	Female	45,XX,-20[3]/46,XX[11, Mut: FLT3 ITD low	7+3/Midostaurin	CR	intermediate	4
31	71	Female	46XX, Mut: TET2, CTNNA1,NPM1, ATM	Vyxeos	CRi	favorable	3
32	74	Male	46XY, Mut: TET2, KAT6A, IDH2, ASXL1, U2AF1	Decitabine/venetoclax	CRi	poor	1

CR: complete remission; Cri: complete remission with incomplete count recovery; ELN: European LeukemiaNet; FLAG: a combination of Fludarabine, Cytarabine, Idarubicin and G-CSF; HiDAC: high-dose cytarabine; ida: idarubicin; Mut: mutations; NR: non-responders, 7+3: a course of 7 days of standard-dose cytarabine, and 3 days of an anthracycline antibiotic.

Table S2. Antibody panel.

Tube	Marker	Fluorophore/Channel	Clone	Manufacturer
T and B cells 1	CD62L	FITC	DREG-56	Biolegend
	PD-1 (Nivolumab/anti-IgG4)	PE	Nivolumab/HP6025	FCCC Pharmacy/Southern Biotech
	CD69	APC	FN50	Biolegend
	CD39	PerCP/Cy5.5	TU66	BD
	CD4	AF 700	OKT4	Biolegend
	CD8	Pacific Blue	HIT8a	Biolegend
	CD3	Cy7/PE	UCHT1	Biolegend
	CD56	BUV395	NCAM16.2	BD
	CD45	APC-H7	2D1	BD
	PI	PerCP	N/A	N/A
	CD19	QDOT605	HIB19	Biolegend
	CD45RA	BV510	HI100	BD
T and B cells 2	CD62L	FITC	DREG-56	Biolegend
	2B4	PE	C1.7	Biolegend
	TIGIT	PerCP/Cy5.5	A15153G	Biolegend
	CD4	AF 700	OKT4	Biolegend
	CD8	Pacific Blue	HIT8a	Biolegend
	CD3	Cy7/PE	UCHT1	Biolegend
	CD56	BUV395	NCAM16.2	BD
	CD45	APC-H7	2D1	BD
	PI	PerCP	N/A	N/A
	CD19	QDOT605	HIB19	Biolegend
	CD45RA	BV510	HI100	BD
T and B cells FMT	CD62L	FITC	DREG-56	Biolegend
	IgG4/anti-IgG4	PE	HP6025	Southern Biotech
	IgG1k	APC	P3.6.2.8.1	eBioscience
	IgG2	PerCP/Cy5.5	MOPC-173	Biolegend
	CD4	AF 700	OKT4	Biolegend
	CD8	Pacific Blue	HIT8a	Biolegend
	CD3	Cy7/PE	UCHT1	Biolegend
	CD56	BUV395	NCAM16.2	BD
	CD45	APC-H7	2D1	BD
	PI	PerCP	N/A	N/A
	CD19	QDOT605	HIB19	Biolegend
	CD45RA	BV510	HI100	BD
Treg and NK cells	CD56	FITC	NCAM16.2	BD
	CD70	PE	113-16	Biolegend
	CD127	APC	eBioRDR5	eBioscience
	CD16	PerCP/Cy5.5	3G8	BD
	CD4	AF 700	OKT4	Biolegend
	CD8	Pacific Blue	HIT8a	Biolegend
	CD25	Cy7/PE	BC96	Biolegend
	CD27	BUV737	L128	BD
	CD45	APC-H7	2D1	BD
	PI	PerCP	N/A	N/A
	CD3	BV510	UCHT1	BD
NK cells 1	DNAM1	FITC	11A8	Biolegend
	NKp44	PE	P44-8	Biolegend
	NKp80	APC	5D12	Biolegend
	KIR2DL1/S1/S3/S5	PerCP/Cy5.5	HP-MA4	Biolegend

	NKG2C	AF 700	134522	R&D
	CD57	Pacific Blue	HCD57	Biolegend
	KLRG1	Cy7/PE	14C2A07	Biolegend
	CD56	BUV395	NCAM16.2	BD
	CD45	APC-H7	2D1	BD
	PI	PerCP	N/A	N/A
	LAG-3	QDOT605	T47-530	BD
	CD3	BV510	UCHT1	BD
NK cells 2	NKp46	FITC	9E2	Biolegend
	NKG2A	PE	REA110	Miltenyi Biotec
	NKG2D	APC	1D11	Biolegend
	KIR2DL2/L3	PerCP/Cy5.5	DX27	Biolegend
	LILRB1	AF 700	292305	R&D
	KIR3DL1	Pacific Blue	DX9	Biolegend
	NKp30	Cy7/PE	AF29-4D12	Invitrogen
	CD56	BUV395	NCAM16.2	BD
	CD45	APC-H7	2D1	BD
	PI	PerCP	N/A	N/A
	CD3	BV510	UCHT1	BD
Intracellular	CD56	FITC	NCAM16.2	BD
	CD62L	PE	DREG-56	Biolegend
	FoxP3	APC	236A/E7	Invitrogen
	CD4	PerCP/Cy5.5	OKT4	Biolegend
	Ghost Dye	AF 700	N/A	Tonbo
	CD8	Pacific Blue	HIT8a	Biolegend
	CTLA-4	Cy7/PE	L3D10	Biolegend
	Ki67	BUV395	B56	BD
	CD3	BUV737	UCHT1	BD
	CD45	APC-H7	2D1	BD
	CD45RA	BV510	HI100	BD
Intracellular FMT	CD56	FITC	NCAM16.2	BD
	CD62L	PE	DREG-56	Biolegend
	Perforin	APC	dG9	Biolegend
	CD4	PerCP/Cy5.5	OKT4	Biolegend
	Ghost Dye	AF 700	N/A	Tonbo
	CD8	Pacific Blue	HIT8a	Biolegend
	IgG1k	Cy7/PE	P3.6.2.8.1	eBioscience
	IgG1	BUV395	X40	BD
	CD3	BUV737	UCHT1	BD
	CD45	APC-H7	2D1	BD
	CD45RA	BV510	HI100	BD
BLIMP-1	CD62L	FITC	DREG-56	Biolegend
	BLIMP-1	PE	C14A4	Cell Signaling Technology
	FoxP3	APC	236A/E7	Invitrogen
	CD4	AF 700	OKT4	Biolegend
	CD8	Pacific Blue	HIT8a	Biolegend
	CD3	Cy7/PE	UCHT1	Biolegend
	CD56	BUV395	NCAM16.2	BD
	CD45	APC-H7	2D1	BD
	CD19	QDOT605	HIB19	Biolegend
	CD45RA	BV510	HI100	BD
BLIMP-1 FMO	CD62L	FITC	DREG-56	Biolegend
	IgG	PE	DA1E	Cell Signaling Technology
	FoxP3	APC	236A/E7	Invitrogen

CD4	AF 700	OKT4	Biolegend
CD8	Pacific Blue	HIT8a	Biolegend
CD3	Cy7/PE	UCHT1	Biolegend
CD56	BUV395	NCAM16.2	BD
CD45	APC-H7	2D1	BD
CD19	QDOT605	HIB19	Biolegend
CD45RA	BV510	HI100	BD

AF 700—Alexa Fluor 700, BD—Becton Dickinson Company, FCCC—Fox Chase Cancer Center, PI—propidium iodide.
